# Complement C3 From Astrocytes Plays Significant Roles in Sustained Activation of Microglia and Cognitive Dysfunctions Triggered by Systemic Inflammation After Laparotomy in Adult Male Mice

**DOI:** 10.1007/s11481-024-10107-z

**Published:** 2024-03-01

**Authors:** Ying Chen, John Man-Tak Chu, Gordon Tin-Chun Wong, Raymond Chuen-Chung Chang

**Affiliations:** 1 Department of Anaesthesiology, School of Clinical Medicine, LKS Faculty of Medicine, The University of Hong Kong, K4-24, K Block, Queen Mary Hospital, 102 Pokfulam Road, Pokfulam, Hong Kong SAR, China; 2 Laboratory of Neurodegenerative Diseases, School of Biomedical Sciences, LKS Faculty of Medicine, The University of Hong Kong, L4-49, Laboratory Block, Faculty of Medicine Building, 21 Sassoon Road, Pokfulam, Hong Kong SAR, China; 3 State Key Laboratory of Brain and Cognitive Sciences, The University of Hong Kong, Pokfulam, Hong Kong SAR, China

**Keywords:** Neuroinflammation, Complement C3, Microglia, Astrocyte, Laparotomy

## Abstract

Aberrant activation of complement cascades plays an important role in the progress of neurological disorders. Complement C3, the central complement component, has been implicated in synaptic loss and cognitive impairment. Recent study has shown that wound injury-induced systemic inflammation can trigger the increase of C3 in the brain. Our previous studies have demonstrated that laparotomy-triggered systemic inflammation could induce neuroinflammation and cognitive dysfunctions. Furthermore, sustained activation of microglia was observed even 14 days after laparotomy, while most of cytokines had returned to basal levels rapidly at the earlier time point. Although we have demonstrated that anti-inflammatory intervention successfully attenuated cognitive dysfunction by preventing increase of cytokines and activation of microglia, how sustained activation of microglia and cognitive dysfunction occur is still a mystery. In this study, we investigated the role of C3 in mediating activation of microglia and cognitive dysfunction by using laparotomy in adult male mouse only as the experimental model of systemic inflammation and AAV9-C3shRNA. Our data observed that laparotomy induced neurotoxic reactive astrocytes with an increase of C3 in the hippocampus. Furthermore, inhibition of C3 by AAV9-C3shRNA prevented synaptic engulfment by microglia and attenuated cognitive dysfunctions after laparotomy. Inhibition of C3 did not modulate activation of astrocytes and expression of various cytokines. Current findings demonstrated that C3 plays significant roles in sustained activation of microglia and cognitive dysfunctions, which suggests that C3 is the valuable molecule target to attenuate in neurological conditions characterised by neuroinflammation and cognitive dysfunction.

**Graphical Abstract:**

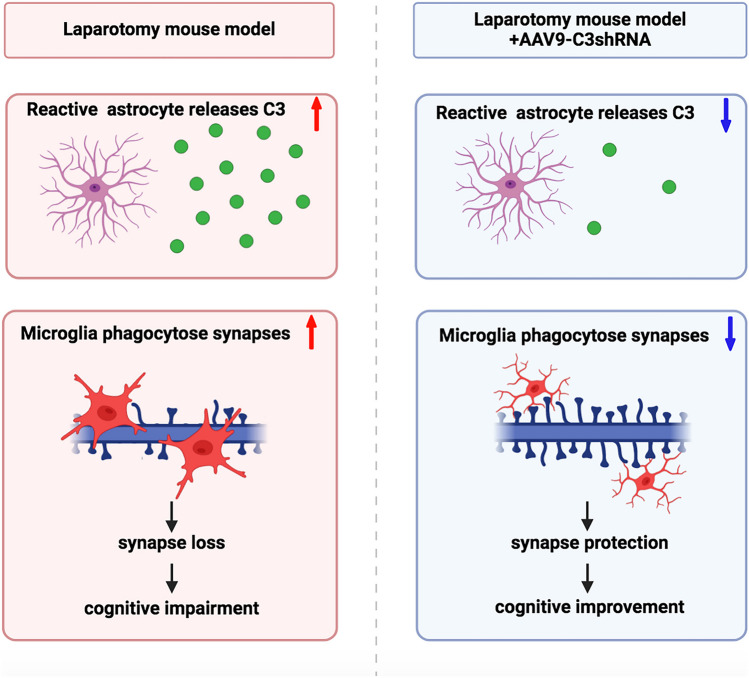

**Supplementary Information:**

The online version contains supplementary material available at 10.1007/s11481-024-10107-z.

## Introduction

The complement system is essential for our immune responses but its function in the CNS spans beyond that of immunity and includes neuroprotection against harmful stimuli and neuroinflammatory responses (Schartz and Tenner 2020; Chen et al. 2022b). Nevertheless, aberrant responses from the complement system exacerbate neurological symptoms and accelerate the development of neurodegenerative diseases (Propson et al. 2021; Ziabska et al. 2021; Chen et al. 2022b). C1q and C3 are important complement factors and both of them have been shown to be upregulated in various neurodegenerative diseases and contribute to synaptic elimination (Presumey et al. 2017; Luchena et al. 2018; Rajendran and Paolicelli 2018). C1q, the initiator of the classical complement pathway, is regarded as the main factor to mediate synaptic pruning in the AD brain by primarily activating complement cascades via the classical pathway (Hong et al. 2016; Dejanovic et al. 2018; Luchena et al. 2018). C3 is the central molecule of the complement cascade and is involved in synaptic elimination by mediating microglial phagocytic activity via the CR3 or C3aR pathways (Hong et al. 2016; Vasek et al. 2016; Litvinchuk et al. 2018; Werneburg et al. 2020). Furthermore, a mouse model of multiple sclerosis with increase of C1q and C3 in the brain has shown that knock-out of C3 protected against synaptic loss, while little improvement was found in C1q knock-out mice (Hammond et al. 2020). It suggests that C3 plays a determinate role of synaptic loss in neurological conditions. A recent study revealed that C3 is increased in the hippocampus after tibial fracture (Xiong et al. 2018), which demonstrated that systemic inflammation triggered by wound injury can induce release of C3 in the central nervous system (CNS). Our previous studies have shown that laparotomy-triggered systemic inflammation can induce neuroinflammation and cognitive dysfunctions. Furthermore, sustained activated morphology of microglia was observed even 14 days after laparotomy, although majority of pro-inflammatory cytokines have returned to basal levels (Huang et al. 2018b). Besides, we found activation of astrocytes in this model. While it is unclear of how sustained activation of microglia and cognitive dysfunctions occur, we hypothesize that C3 fills this information gap and mediates these two pathological events.

Astrocytes being the most abundant glial cell in the CNS, play important roles in maintaining CNS homeostasis, supplying energy to neurons, recycling neurotransmitters, mediating synapse functions, and controlling cerebral blood flow (Dallérac and Rouach 2016; Mächler et al. 2016). However, they can also be agents of harm when they become reactive in response to adverse stimuli, altering their normal functions and contributing to prolonged neuroinflammation (Ben Haim et al. 2015; Liddelow et al. 2017; Clarke et al. 2018; Hou et al. 2020). It has been proposed that reactive astrocytes can be divided into neurotoxic and neurotrophic subtypes, denoted as A1 and A2 astrocytes, respectively. They have their own distinct cell markers, transcriptional profiles, and functions (Ben Haim et al. 2015; Liddelow et al. 2017). When exposed to specific proinflammatory factors: IL-1α, TNF-α and C1q, astrocytes can turn to A1 neurotoxic phenotype and further contribute to cognitive deficits in Alzheimer’s disease (AD) (Liddelow et al. 2017). A1 neurotoxic astrocytes were proposed to be identified by 12 genes (H2.T23, Serping1 H2.D1, Ggta1 Ligp1, Gbp2, Fbln5, Ugtta, Fkbp5, Psmb8, Srgn and Amigo2) with 13 pan-reactive genes (Lcn2, Steap4, S1pr3, Timp1, Hspb1, Cxcl10, Cd44, Osmr, Cp, Serpina3n, Aspg, Vim and Gfap). Also, C3 was reagarded as the A1 marker, because of its upregulation in A1 astrocytes but no expression in A2 astrocytes that was induced by ischemic stroke (Liddelow et al. 2017). A1 astrocytes no longer promoted neuronal survival, outgrowth and synaptogenesis, but induced death of neurons and oligodendrocytes (Liddelow et al. 2017). This type of astrocytes have been observed in perioperative neurocognitive disorders (PNDs) (Li et al. 2020). However, little is known about the impact of reactive astrocytes on laparotomy.

PNDs is a common postsurgical complications characterized by cognitive decline, the incidence rate ranges from 17 to 43% and it is even higher in aged population (Mahanna-Gabrielli et al. 2019). Patients with PNDs display cognitive dysfunctions, lower quality of life, and even increased mortality (Borozdina et al. 2018; Mahanna-Gabrielli et al. 2019). All these problems can result in extended hospital stay and reduced independence in activities of daily living (Mahanna-Gabrielli et al. 2019). Laparotomy is an experimental model for PNDs. While laparotomy has clinical relecance, this experimental model also provides excellent opportunity to investigate how systemic inflammation induces neuroinflammation and cognitive dysfunction. It has been generally considered that neuroinflammation contributes to the development of neurodegenerative diseases, mental disorders, and PNDs (Heppner et al. 2015; Safavynia and Goldstein 2018; Gilhus and Deuschl 2019; Luo et al. 2019; Yang et al. 2020). Our previous studies have shown that surgery- or LPS-induced systemic inflammation triggered the occurrence of neuroinflammation, which further led to cognitive impairment (Chu et al. 2018; Huang et al. 2018a). Postoperative neuroinflammatory responses include rapid but transient increases of cytokines, such as IL-1β, TNF-α and IL-6, and relatively long-lasting activation of glia (Chu et al. 2018; Huang et al. 2018b). Among different cytokines, IL-1β plays significant roles in attracting infiltration of monocytes/macrophages into the brain by amputation in zebrafish (Chen et al. 2022a). Furthermore, we have demonstrated that anti-inflammatory pharmacological intervention could successfully attenuate cognitive deficits by preventing increase of cytokines and activation of microglia (Chu et al. 2018; Huang et al. 2018a). As stated above, the factors mediating sustained activation of microglia and cognitive dysfunctions are unclear. Therefore, we investigate whether C3 plays key roles in mediating these two pathological events.

In this study, we observed and identified that laparotomy-induced reactive astrocytes were neurotoxic phenotype with an increase of C3. We further demonstrated that knock-down of C3 by shRNA attenuated cognitive dysfunctions following surgery. Besides, knock-down of C3 prevented engulfment of synapses by microglia and synaptic loss in the hippocampus after laparotomy. Our findings provide evidence showing that component complement C3 can be a key player to sustain activation of microglia and cognitive dysfunctions.

## Materials and Methods

### Mice and Treatment

#### Mice

Adult male C57/B mice between 12–16 weeks of age were obtained from The Centre for Comparative Medicine Research, the University of Hong Kong, which is accredited by the Association for Assessment and Accreditation of Laboratory Animal Care International. All mice were housed in temperature-controlled room under a 12/12 h light/dark cycle and have free access to food and water. All experimental protocols, handling procedures and research ethics were approved by the Faculty Committee on the Use of Live Animals in Teaching and Research (CULATR, Ref. No. 5147–19).

#### Experimental Design

In the first experiment, the mice were randomly divided into three groups: neither anesthesia nor surgery as control (CON), sevoflurane anesthesia only (SEVO) and laparotomy under sevoflurane anesthesia (LAP). In the second experiment, the mice were randomly divided into 4 groups: sevoflurane anesthesia with control AAV9 vector (SEV + AAV9-GFP), sevoflurane anesthesia with anti-C3 shRNA vector (SEV + AAV9-C3shRNA), laparotomy with control AAV9 vector (LAP + AAV9-GFP) and laparotomy with anti-C3 shRNA AAV9 vector (LAP + AAV9-C3shRNA).

#### Laparotomy Model

All surgical procedures were performed on a heating pad with the frequency of respiration and the color of the paws monitored continuously, which has been described before with modifications (Huang et al. 2018a). Buprenorphine (0.1 mg/kg) was injected subcutaneously 30 min before surgery for analgesia. General anesthesia was then induced and maintained using 3% sevoflurane in 1 L/min of oxygen. Once anesthesia was established, a 2.5 cm longitudinal incision was made in the midline of the abdomen. Approximately 10 cm of intestine was taken out of the abdominal cavity and gently rubbed for 2 min. After a further minute, the bowel loops were replaced into the abdominal cavity. Absorbable sutures were used to close the muscle layers of the incision and nonabsorbable sutures were used for the skin. After the mouse recovered from anesthesia, it was transferred to a clean cage with soft bedding. The whole surgical procedure required approximately 25 min. Buprenorphine was given for a further three days for post-operative analgesia. The sevoflurane group only received 3% sevoflurane for 25 min in the same condition as the laparotomy group.

#### Stereotaxic Injection

Buprenorphine (0.1 mg/kg) was injected 30 min before surgery, and general anesthesia was induced by a ketamine/xylazine cocktail (ketamine 80–100 mg/kg + xylazine 5–10 mg/kg). Each mouse was fixed onto the stereotaxic apparatus and was injected with 2 ul of AAV vector (2–3 × 10^12^ v.g/ml, 1 ul/min) into lateral ventricle, using a 2 ul Hamilton glass syringe (Hamilton, series 7000). The coordinates for lateral ventricle injection were determined as follows: AP(Bregma): -0.6; ML: ± 1.3; DV(Skull): + 3.0.

### Construction of the Anti-C3 shRNA AAV9 Vector

The anti-C3 shRNA was cloned downstream of the U6 promoter in the AAV9 vector plasmid (Vigenbio, China) and a green fluorescent protein (GFP) sequence was inserted following the anti-C3 shRNA sequence to indicate transfection and titer assay by RT-PCR. In the control plasmid, a similar length but nonsense sequence was inserted instead of the anti-C3 shRNA sequence after the U6 promoter before the GFP sequence. The anti-C3 shRNA sequence: 5’-GCACTATGCACAACTCCAACATTCAAGAGATGTTGGAGTTGTGCATAGTGCTTTTT-3’.

The anti-C3-shRNA AAV9 vector was constructed according to triple plasmid protocols. The AAV9 packaging pAdDeltaF6 (Addgene, USA), helper plasmid pAAV2/9n (Addgene, USA) and pAAV-anti-C3-shRNA plasmid were transfected at a ratio of 1:1:1 into HEK293T cells when their confluence reached approximately 70%. At 6 h after post-transfection, the medium was replaced with fresh culture media containing 5% fetal bovine serum and the cells were further cultured for 96 h at 37^0^C. The cells were then harvested and centrifuged at 2000 g. The viral solution was extracted and purified using the AAVpro purification kit (Takara, Japan, Cat. # 6675). The titers of control AAV9 vector (AAV9-GFP) and anti-C3 shRNA AAV9 vector (AAV9-C3shRNA) were quantified by real-time PCR using the forward GFP primer 5’-GCA TCG ACT TCA AGG AGG AC-3’ and reverse GFP primer 5’- GAA CTC CAG CAG GAC CAT GT-3’. The knock-down efficiency of AAV9-C3shRNA was verified in BV2 cultured cells (Suppl. Fig. 2A-B).

### Statistical Analysis

All data were presented as mean ± SEM, each experimental group included at least 3 independent replicates. GraphPad Prism (version7.0) was used for statistical analyses. For continuous data, statistical significance was evaluated by two-tailed Student’s t test to compared two groups, one-way ANOVA followed by Tukey’s multiple comparison post hoc test to compare multiple groups, two-way ANOVA followed by Bonferroni’s multiple comparison post hoc test to compare both C3 knock down and laparotomy effects, and repeated measure ANOVA followed by Bonferroni’s multiple comparison post hoc test for body weight changes after laparotomy. For discrete data such as the number of errors in the behavioral tests, Kruskal–Wallis test followed by Dunn’s multiple comparisons test was used. * P < 0.05, **P < 0.01, ***P < 0.001.

All other information are in Supplementary Materials.

## Results

### Laparotomy Induced Cognitive Decline in WT Adult Mice

**Fig. 1:**
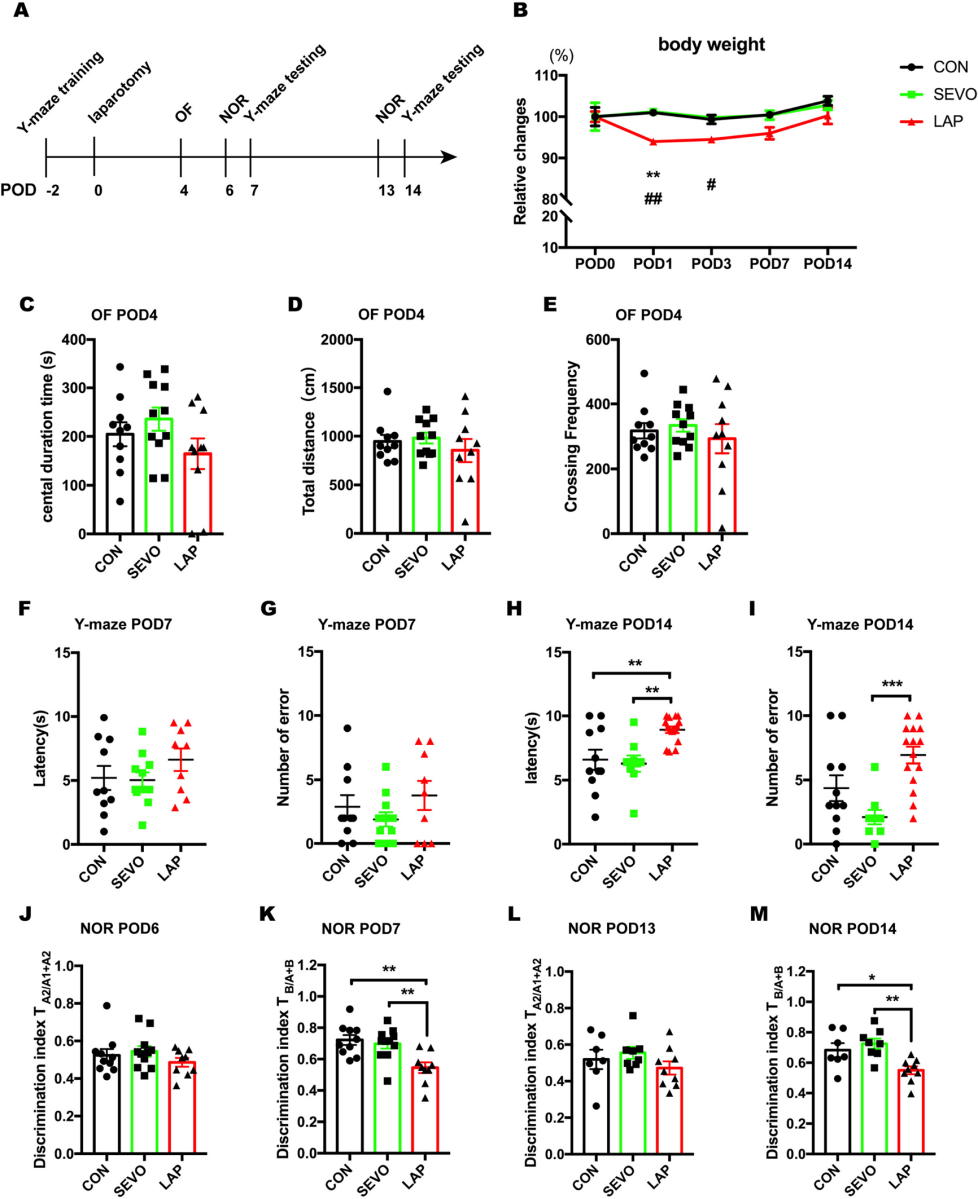
Laparotomy induced learning and memory decline in adult WT mice. **A**: Schema of the experimental design. **B**: The changes in body weight at different timepoints during the postoperative period. Two-way repeated ANOVA with Tukey’s multiple comparison test with n = 13–15 mice per group, CON vs LAP: ** P < 0.01, SEVO vs LAP: # P < 0.05, ## P < 0.01. **C-E:** Open field test on POD4, the duration time in the central area (**C**), Total distance (cm) during 10 min observation (**D**), the frequency of grid crossing (**E**), CON: n = 10, SEVO: n = 11, LAP: n = 10. **F-I**: Y-maze test on PODs 7 and 14, including: the latency on POD 7 (**F**), the number of errors on POD 7 (**G**), CON: n = 10, SEVO: n = 11, LAP: n = 9; the latency on POD14 (**H**), the number of errors on POD14 (**I**), CON: n = 11, SEVO: n = 9, LAP: n = 15. One-way ANOVA with Tukey’s multiple comparison test was applied to the analysis of latency, Kruskal–Wallis test with Dunn’s multiple comparisons test was applied to the number of errors. **J-M**: NOR test on PODs 7 and 14, including: discrimination index of two similar objects (A1 and A2) in the familiarization phase on POD6 (**J**); discrimination index of novel object (**B**) in the testing phase on POD7 (**K**), CON: n = 10, SEVO: n = 11, LAP: n = 9; discrimination index of two similar objects (A1 and A2) in the familiarization phase on POD13 (**L**), discrimination index of novel object (**B**) in the testing phase on POD14 (**M**), CON: n = 7, SEVO: n = 8, LAP: n = 9. One-way ANOVA with Tukey’s multiple comparison test. Data is presented as mean ± SEM, * P < 0.05, **P < 0.01, ***P < 0.001. POD: post-operative day, Y-maze test = forced alternation Y-maze test, NOR test = novel object recognition test, CON = control, SEVO = sevoflurane, LAP = laparotomy

Firstly, we applied laparotomy on adult male WT mice under sevoflurane anesthesia and assessed cognitive functions on POD 7 and 14 by using forced Y-maze and NOR tests (Fig. 1A). Mice that underwent laparotomy showed a significant reduction in the relative body weight on the early stage of postoperative period while there were little changes to the mice exposed to sevoflurane anesthesia only (Fig. 1B). Since surgical trauma may induce anxiety or impair locomotor ability, and both of them can affect the performance on behavioral tests, we conducted the OF test on POD 4 to evaluate their presence. No significant difference was found in the central duration time, total distance, and grid crossing frequency among 3 groups, indicating neither sevoflurane anesthesia and laparotomy induced anxiety-like behavior or locomotor impairment (Fig. 1C-E). To further determine whether laparotomy affects the cognitive function, we assessed learning and memory used by forced alternation Y-maze and NOR tests on both POD 7 and 14. Mice that underwent laparotomy showed a significant impairment in recognizing the white safe arm with a longer latency time and larger number of errors than the SEVO group on POD 14 but not POD 7 (Fig. 1F-I). Furthermore, mice from the LAP group showed less interaction with the novel object when tested on both POD 7 and 14, and onwards with no object or location preference in the advance familiarization session (Fig. 1J-M). Results from these behavioral tests suggest that cognitive impairment is present at least between these 2 time points after surgery, while sevoflurane anesthesia had little impact on cognitive performance.

### Neuroinflammatory Responses With Prolonged Activation of Glia Occurred in the Postsurgical Hippocampus

**Fig. 2:**
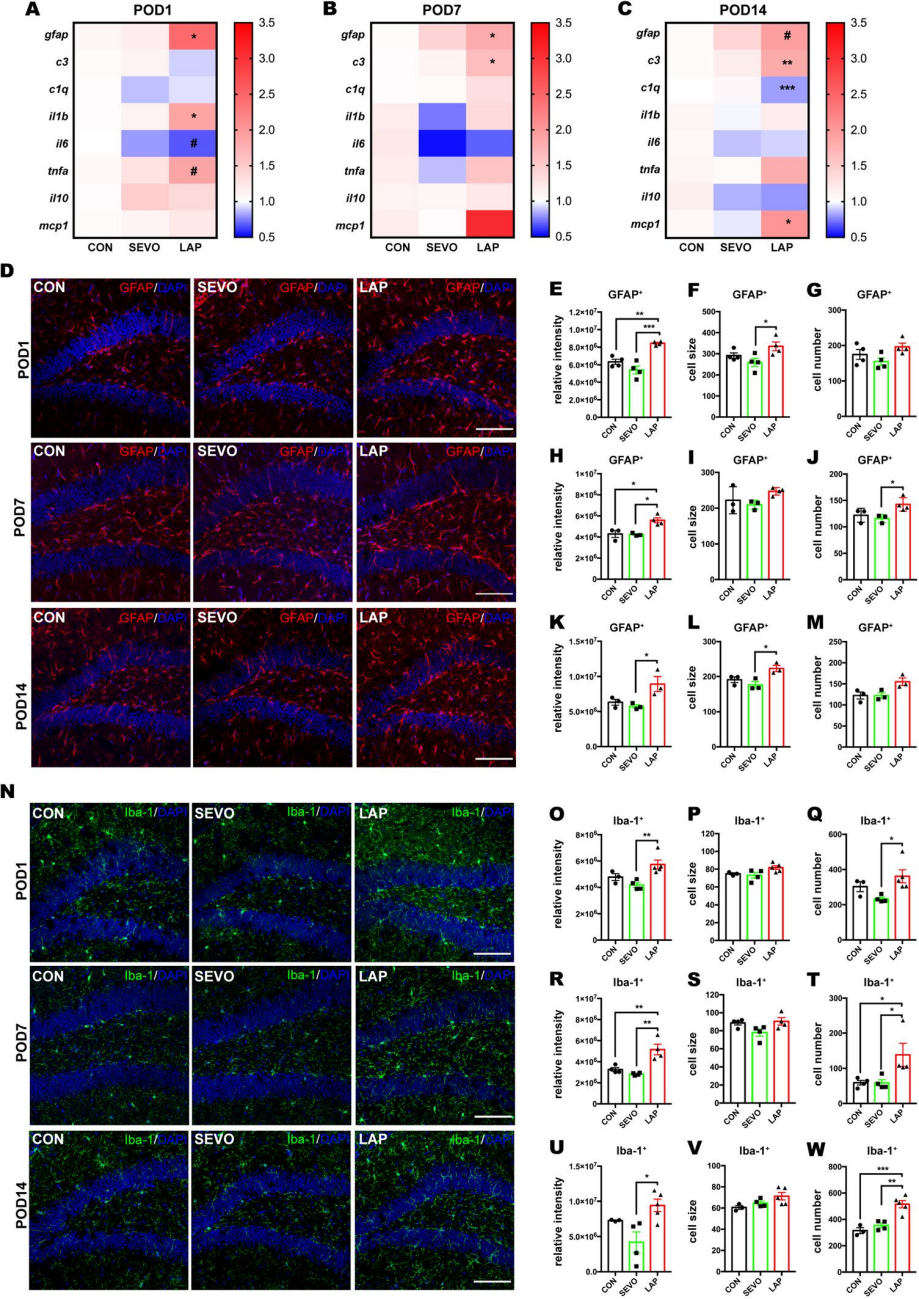
Neuroinflammation with cytokine increase and activation of glia in postsurgical mice.** A-C:** heatmap of cytokine mRNA expression on POD 1 (**A**), POD7 (**B**), and POD14 (**C**)*,* * P < 0.05*,* **P < 0.01, ***P < 0.001 for LAP versus SEVO, # P < 0.05 for LAP versus CON.** D**: representative confocal images show astrocytic marker GFAP staining on PODs 1, 7 and 14, scale bar: 100um. **E–G**: histograms showed GFAP^+^ signals quantification on POD1 including relative intensity (**E**), average cell size (**F**) and total cell number (**G**). **H-J**: histogram shows GFAP^+^ signals quantification on POD 7 including relative intensity (**H**), average cell size (**I**) and cell number (**J**). **K-M**: histogram shows GFAP^+^ signals quantification on POD 14 including relative intensity (**K**), average cell size (**L**) and cell number(**M**). **N**: representative confocal images show microglial marker Iba-1 staining on PODs 1, 7, and 14, scale bar: 100 um. **O-Q**: histogram shows Iba-1^+^ signals quantification on POD1 including relative intensity (**O**), average cell size (**P**) and total cell number (**Q**). **R-T**: histogram shows Iba-1^+^ signals quantification on POD 7 including: relative intensity (**R**), average cell size (**S**) and total cell number (**T**). **U-W**: histogram shows Iba-1^+^ signals quantification on POD 14 including: relative intensity (**U**), average cell size (**V**) and total cell number (**W**). The representative images were from hippocampal DG area. The average of DG, CA1 and CA3 areas were used for statistical analysis. Data is presented as mean ± SEM, IF staining analysis was used One-way ANOVA with Tukey’s multiple comparison test with n = 3–5 mice per group. * P < 0.05, **P < 0.01, ***P < 0.001. POD = post-operative day, CON = control, SEVO = sevoflurane, LAP = laparotomy

Neuroinflammation has been shown to contribute to postoperative cognitive decline (Safavynia and Goldstein 2018; Luo et al. 2019; Yang et al. 2020). The postsurgical mice have higher levels of pro-inflammatory factors and cellular activation in the hippocampus or the frontal cortex (Luo et al. 2019; Yang et al. 2020). To verify these findings, we measured the change of pro-inflmmatory factors in the postsurgical hippocampus on PODs 1, 7 and 14, especially those rodents that were subjected to behavior tests on PODs 7 and 14. In this study, the mRNA levels of several pro-inflammatory factors showed significant changes at different time points. On POD 1, IL-1β and TNF-α were increased but IL-6 was decreased in the LAP group (Fig. 2A). On POD7, C3 were significantly increased in the LAP group (Fig. 2B). On POD 14, C3 and MCP-1 were significantly increased but C1q was significantly decreased in the LAP group (Fig. 2C). We noticed that cytokines IL-1β, TNF-α and IL-6 changes were rapid but transient, while C3 upregulation was delayed but prolonged, a pattern that concur more with the time course of cognitive deficits.

In addition, GFAP and Iba-1 staining showed prolonged activation of astrocytes and microglia after laparotomy. In the postsurgical hippocampus, GFAP^+^ astrocytes presented with higher intensity on POD 1, 7 and 14, with more extensive cell size on POD1 and 14 and increased cell number on POD 7 (Fig. 2D-M). Similarly, Iba-1^+^ microglia in the postsurgical hippocampus presented with a higher Iba-1 intensity and increased cell number on POD 1, 7, and 14, but the cell size did not show any difference among three groups (Fig. 2N-W).

### RNA-sequencing Revealed that Postsurgical Hippocampal Astrocytes Had Distinct Transcriptional Profiles

**Fig. 3:**
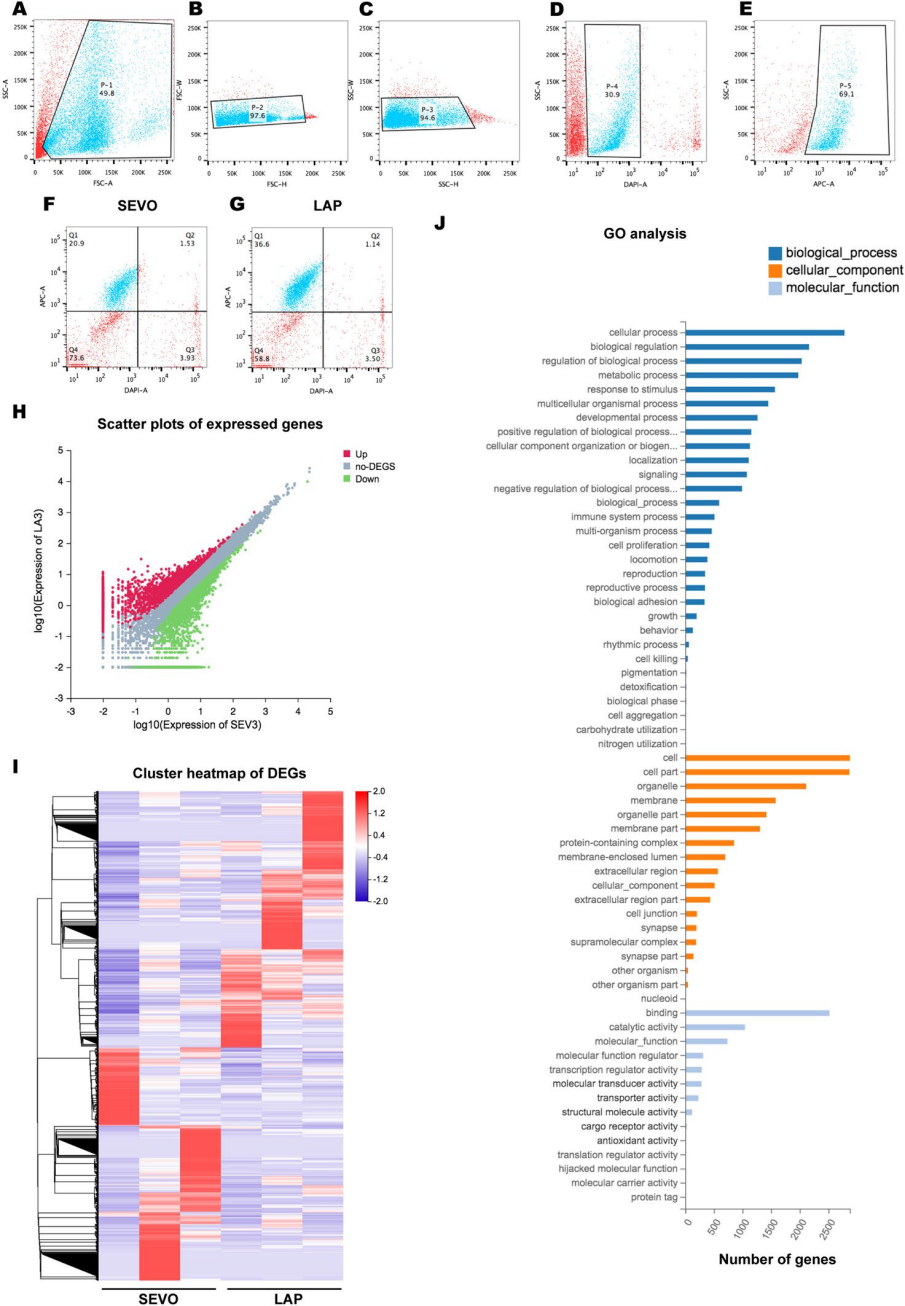
Transcriptional characteristics of hippocampal astrocytes from the postsurgical mice. **A-G:** representative dot plot showing FACS gating strategy for live GLAST^+^ astrocytes, P-1/P-2/P-3 shows single cell gating (**A-C**), P-4 under P-3 shows DAPI^−^ live cells (**D**), P5 under P-4 shows GLAST^+^ cells (**E**), Q1 quadrant shows 20.9% DAPI^−^/GLAST^+^ live astrocytes in SEVO mice (**F**), and 36.5% DAPI^−^/GLAST^+^ live astrocytes in LAP mice (**G**). **H**: scatter plot of differentially expressed genes (DEGs), red represents upregulated genes, green represents downregulated genes, and grey represents non-regulated genes, |log2 (foldchange)|≥ 1 and Q value ≤ 0.05 are used as screening threshold. **I**: the cluster heatmap of all DEGs. **J**: histogram shows GO functional classification analysis. Data is presented as mean ± SEM, * P < 0.05. SEVO = sevoflurane, LAP = laparotomy

Emerging evidence indicated that reactive astrocytes could turn to A1 neurotoxic phenotype and accelerated the progression of neurodegenerative disease by releasing active molecules, such as C3 (Liddelow et al. 2017). Therefore, we isolated live GLAST^+^ astrocytes from the postsurgical hippocampus using flow cytometry (Fig. 3A-G) and performed RNA sequencing and differentially expressed genes (DEGs) analysis on these astrocytes for a more comprehensive understanding of gene expression profiles after surgery. The results showed that there were 3777 DEGs over a total of 16,429 detected genes based on the comparison between the SEVO and LAP groups (Fig. 3H), and the distribution was shown in the cluster heatmap of DEGs (Fig. 3I). The following gene ontology (GO) analysis revealed the enrichment of DEGs in three major categories: biological process, cellular component and molecular functions. It showed that most of DEGs were enriched in cellular process, biological regulation, regulation of biological process and metabolic process under “biological process” category, most of DEGs belonged to cell and cell part under “cellular component” category, and majority of DEGs significantly belonged to binding when referred to “biological functions” (Fig. 3J).

Thus, the significant difference of transcriptional profile from postsurgical astrocytes suggested higher activities in signaling interaction and transduction and the possible involvement of immune responses, which concurred with astrocytic reactivation after surgery.

### Laparotomy-induced Reactive Astrocytes Were A1 Neurotoxic Phenotype With C3 Upregulation

**Fig. 4:**
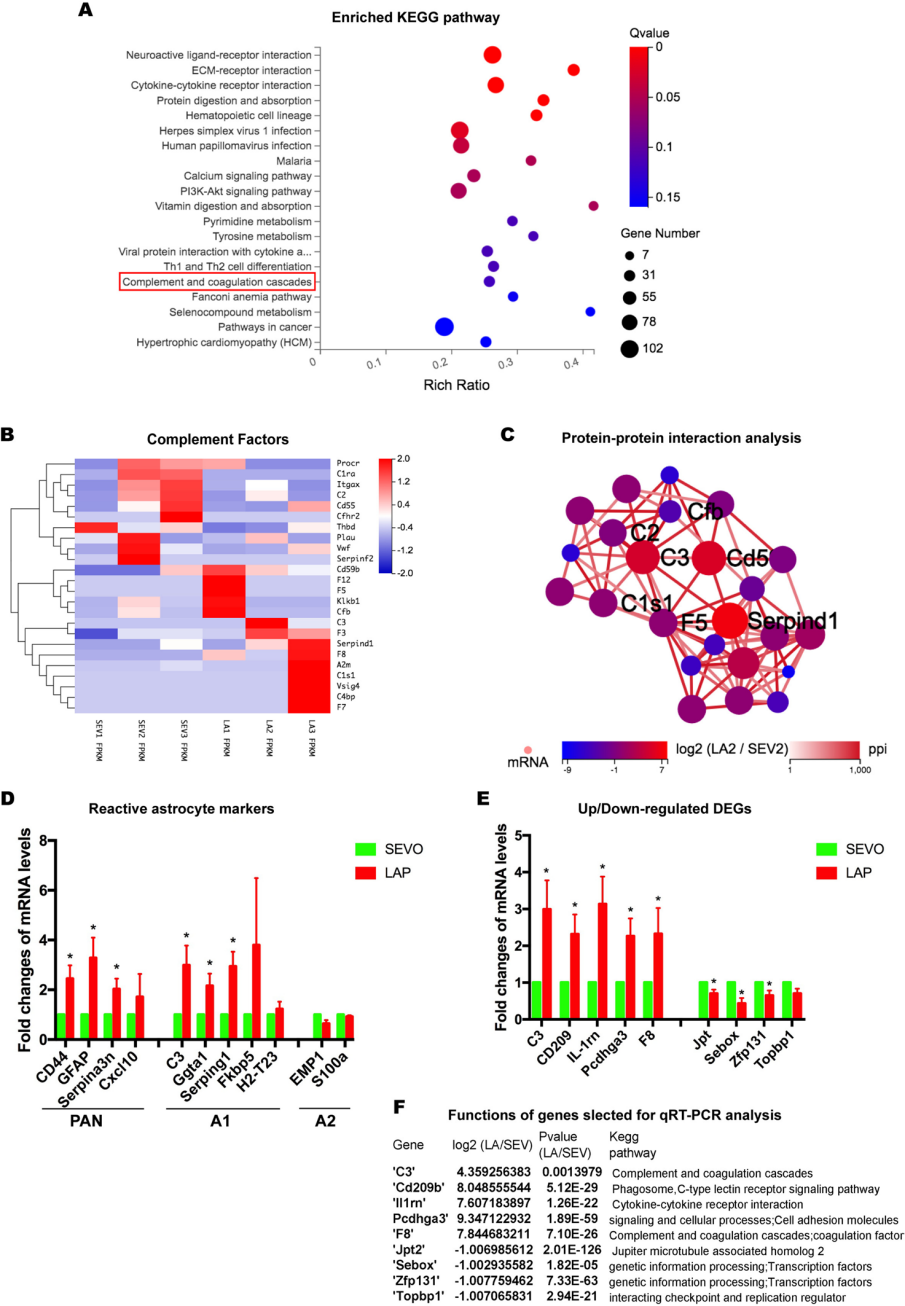
Hippocampal astrocytes from the postsurgical mice were identified as neurotoxic reactive astrocytes with the activation of complement cascades.** A**: the KEGG pathway analysis reveals the top 20 enrichments in the comparison between LAP mice and SEVO mice** B**: heatmap of 24 regulated complement factors, red represents upregulated genes and blue represents downregulated genes*.* **C**: protein–protein interaction network is based on 24 regulated complement factors. **D**: histogram shows relative mRNA levels of reactive astrocytic markers*.* **E**: histogram shows relative mRNA changes of selected genes for validation, n = 6–8 mice per group*.* **F**: the table shows the relative fold changes in RNA-seq analysis and functional properties of selected genes for RT-PCR validation. Data is presented as mean ± SEM. Student’s t test was performed to data analysis. * P < 0.05. SEVO = sevoflurane, LAP = laparotomy

Followed by the GO analysis, we performed the KEGG (Kyoto Encyclopedia of Genes and Genomes) pathway analysis to see potential signaling pathways involved in astrocytic reactivation. Firstly, several pathways related to the immune system were listed in the top 20 enriched KEGG terms, including complement and coagulation cascades, hematopoietic cell lineage, and Th1 and Th2 cell differentiation (Fig. 4A). This list also displayed other pathways involved in signaling interaction and transduction, such as neuroactive ligand-receptor interaction, ECM-receptor interaction, cytokine-cytokine receptor interaction, calcium signaling pathway, and PI3K-Akt signaling pathway, same as metabolism-related pathways that were also affected (Fig. 4A).

Considered that the complement and coagulation cascades was highly involved in astrocytic reactivation, which was also associated with the development of cognitive deficits, we further examined the activation of complement cascades in postsurgical astrocytes. Firstly, the heatmap of complement factors showing the relative expression of complement DEGs with elevation of Cd59b, F12, F5, Klkb1, Cfb, C3, F3, Serpind1, F8, A2m, C1s1, Vsig4, C4bp, and F7 in the LAP group (Fig. 4B). The following protein–protein interaction analysis of complement factors suggested that C3 was one of the central factors in complement activation induced by surgery (Fig. 4C).

Upregulation of C3 and activation of complement in postsurgical reactive astrocytes suggested that surgery could cause the neurotoxic phenotype of reactive astrocytes. Thus, we investigated the changes of reactive astrocyte markers by RT-PCR to further characterize these reactive astrocytes in the postsurgical hippocampal region. Compared to the SEVO group as control, GLAST^+^ astrocytes in the LAP group showed a significant increase in pan-active astrocyte markers (Gfap, Cd44 and Serpina3n) and A1 astrocyte markers (C3, Ggta1, and Serping1), but no change was found in A2 astrocyte markers (Emp-1, S100a), proving that surgery induced reactive astrocytes in the hippocampus was A1 neurotoxic phenotype (Fig. 4D). Besides, we selected 9 genes involved in inflammation or transcriptional activities (Fig. 4F**)**, and measured their expression by RT-PCR to further verify the RNA-seq results (Fig. 4E). These genes exhibited similar changes to the RNA-seq analysis, with 5 genes (C3, Cd209, Il-1rn, Pcdhga3, F8) upregulated and 4 genes (Jpt, Sebox, Zfp131, Topbp1) downregulated **(**Fig. 4E).

Thus, surgery induced reactive astrocytes were identified as the neurotoxic phenotype, featured by the complement activation and C3 upregulation as the central molecular player.

### Astrocytic But Not Microglial C3 Increased in the Hippocampus After Laparotomy

**Fig. 5:**
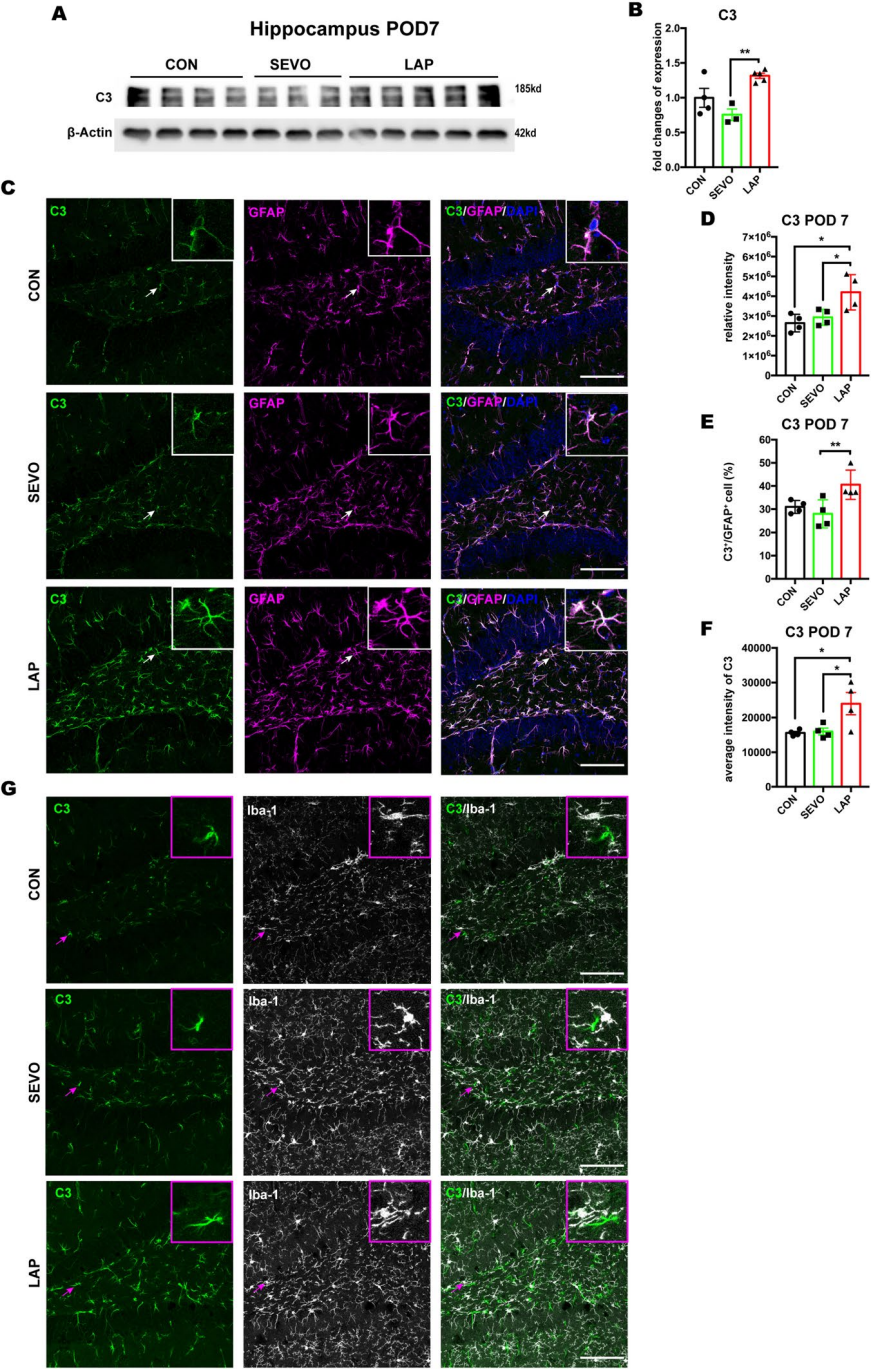
The increase of C3 was observed in astrocytes but not in microglia in the postsurgical hippocampus on POD7.** A-B:** Western blot analysis of C3 protein level in the hippocampus on POD 7, normalized by β-Actin expression, n = 3–5 mice per group. **C:** representative confocal images depict the colocalization of C3 (green) and astrocytic marker GFAP (magenta) staining in the hippocampus on POD7, scale bar: 100um. **D-F:** histograms show the quantified analysis of total C3 intensity (**D)**, the percentage of C3^+^ signal occupied within GFAP^+^ area (**E**), and average C3 intensity within GFAP^+^ cell (**F**), n = 3–4 mice per group. **G**: representative confocal images depict rare colocalization of C3 (green) and microglial marker Iba-1 (gray) in the hippocampus. The representative images were from hippocampal DG area. The average of DG, CA1 and CA3 areas were used for statistical analysis. Data is presented as mean ± SEM. One-way ANOVA with Tukey’s multiple comparison test was used for data analysis, * P < 0.05, **P < 0.01, ***P < 0.001. POD = post-operative day, CON = control, SEVO = sevoflurane, LAP = laparotomy

Recent studies have demonstrated that neurotoxic reactive astrocytes in pathological conditions were able to produce C3, which accelerated the pathogenesis of cognitive impairment in various neurodegenerative diseases. (Liddelow et al. 2017; Clarke et al. 2018; Chen et al. 2022b). The RNA-seq results already showed that the transcription of C3 was increased in surgery-induced A1 astrocytes. Thus, we examined the protein level of C3 and its colocalization in the postsurgical hippocampus to further verify previous findings. Firstly, the results showed that hippocampal C3 protein levels were significantly increased in the LAP group on POD 7, compared to the SEVO group (Fig. 5A-B**,** raw blots seen in Suppl. Fig. 4A). Furthermore, immunofluorescent co-staining of C3 and astrocytic marker GFAP showed that total C3 immunoreactivity in the hippocampus and average intensity of C3 within GFAP^+^ cells were both higher in the LAP group than that in the CON and SEVO groups, with a greater colocalization with GFAP^+^ area in the LAP group on POD 7. This result indicated that C3 was increased in the postsurgical hippocampus and mainly colocalized with GFAP^+^ astrocytes (Fig. 5C-F). However, there was little colocalization of C3 and microglial marker Iba-1 (Fig. 5G). Similar to the change on POD7, C3 protein levels from total hippocampal lysate, total C3 immunoreactivity and average intensity of C3 within GFAP^+^ astrocytes in the hippocampus were increased in the LAP mice on POD14, while the colocalization of C3 with GFAP^+^ area did not show any significant change (Suppl. Fig. 1). Thus, we concluded that surgery induced C3 upregulation in the hippocampus on both POD 7 and 14, which was mainly derived from astrocytes other than microglia, and the changes of astrocytic C3 were related with the change of C3 mRNA levels.

### Inhibition of C3 by AAV9-C3shRNA Attenuated Postoperative Cognitive Impairment

**Fig. 6:**
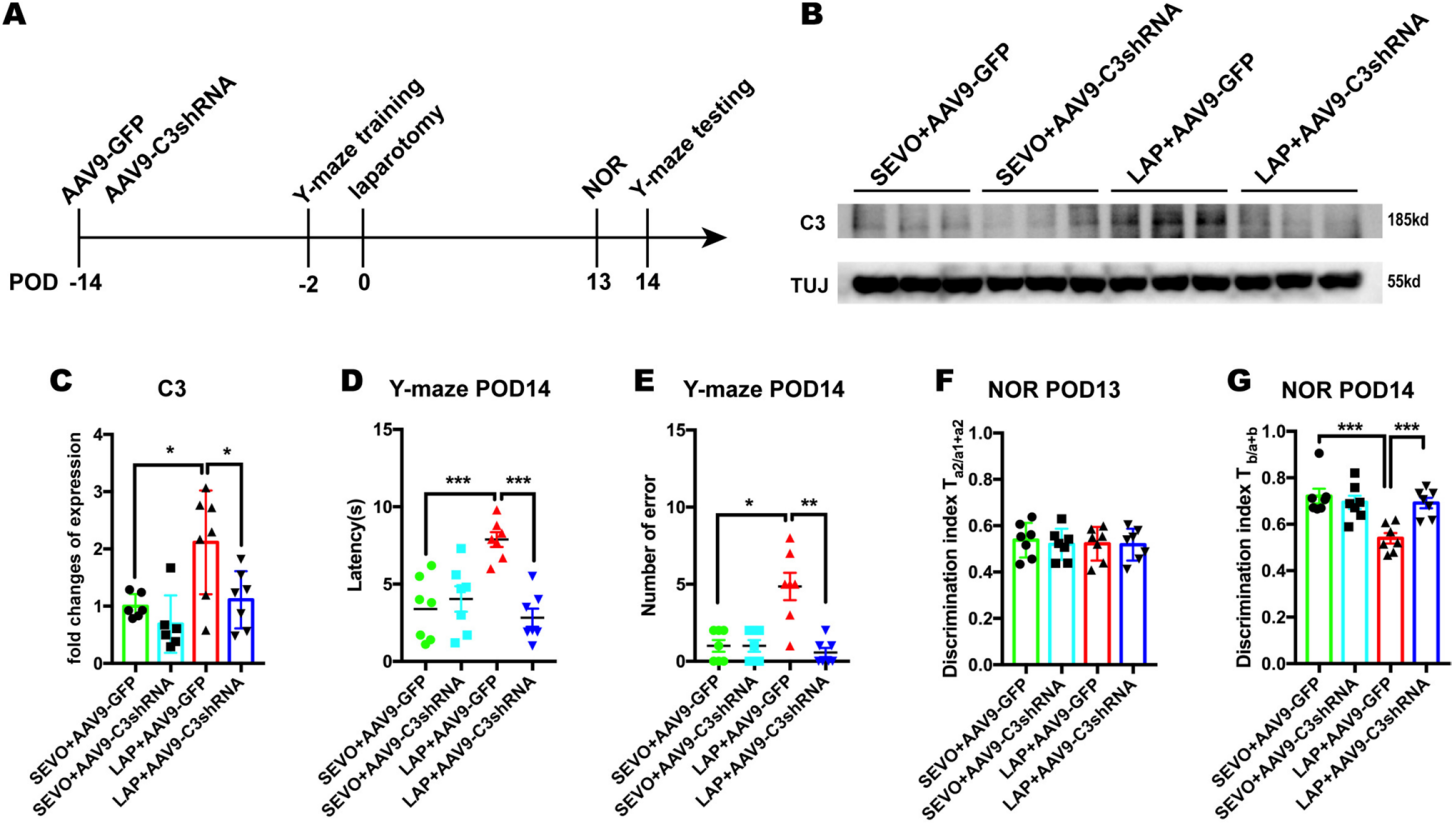
C3 knock-down attenuated post-operative cognitive impairment. **A:** Schema of the experimental design of C3 intervention. **B-C**: Western blot analysis of hippocampal C3 protein level on POD14. Two-way ANOVA with Bonferroni’s multiple comparison test with n = 6–7 mice per group. **D-E**: Y-maze test on POD 14, including: the latency on POD14 (**D**), the number of errors on POD14 (**E**). Two-way ANOVA with Bonferroni’s multiple comparison test was applied to the analysis of latency, Kruskal–Wallis test with Dunn’s multiple comparison test was applied to the number of errors with n = 6–7 mice per group. **F-G**: NOR test on POD 14, including: discrimination index of two similar objects (A1 and A2) on POD13 training (**F**); discrimination index of novel object (**B**) on POD14 NOR testing (**G**). Two-way ANOVA with Bonferroni’s multiple comparison test with n = 6–7 mice per group

To further investigate the role of C3 in cognitive impairment following surgery, we inhibited cerebral C3 by intracerebroventricular injection of AAV9-C3shRNA 14 days before laparotomy and assessed the changes in cognitive testing on POD 14 (Fig. 6A**,** raw blots seen in Suppl. Fig. 4B). The change of body weight showed that laparotomy resulted in significant body weight loss on POD3, while C3 inhibition did not affect body weight of the sevoflurane or laparotomy mice (Suppl. Fig. 2C).The protein levels of hippocampal C3 suggested that AAV9 delivery of anti-C3shRNA successfully suppressed surgery-induced C3 upregulation compared to postsurgical mice treated by AAV9-GFP controls, while not affecting C3 expression in the sevoflurane mice (Fig. 6B-C). Moreover, AAV9-mediated C3 restriction significantly improved cognitive performance of postsurgical mice in both Y-maze and NOR tests compared to AAV9-GFP counterparts. In contrast, we did not find any significant difference between the SEVO + AAV9-GFP and SEVO + AAV9-C3shRNA mice (Fig. 6D-G). The open field test conducted on POD4 showed that no significant difference was found in the central duration time, total distance, and grid crossing frequency among 4 groups, indicating that neither anxiety-like behavior nor locomotor impairment was inducted by laparotomy or C3 inhibition (Suppl. Fig. 2D-F). These behavioral data suggested that the cognitive alternation during postoperative period highly correlated to C3 activation.

### Inhibition of C3 By AAV-C3shRNA Did Not Modulate Reactive Astrocytes and Expression of Cytokines

**Fig. 7:**
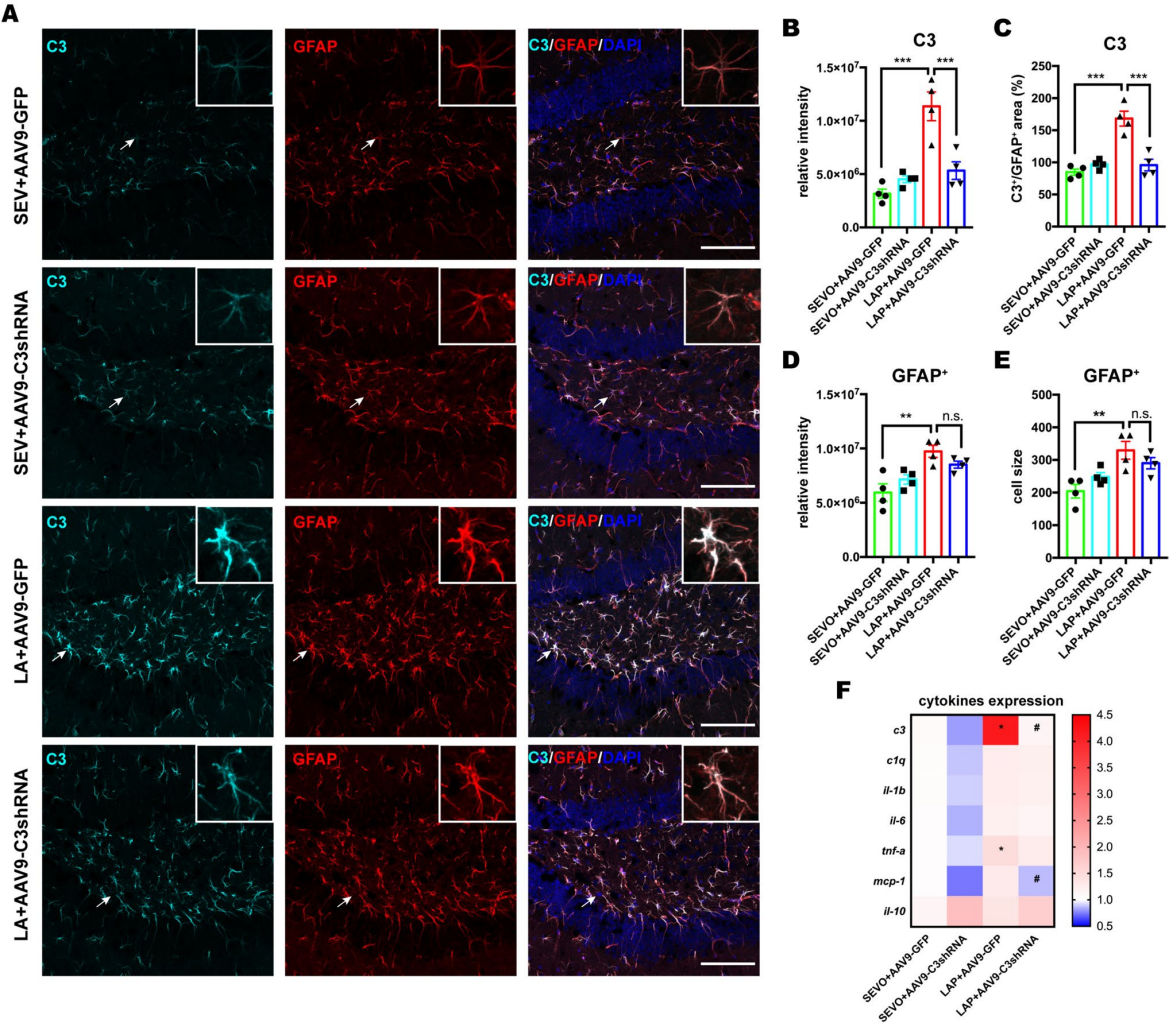
C3 knock-down had limited effects on activation of astrocytes and expression of cytokines.** A**: representative confocal images depicting colocalization of C3 (cyan) and astrocytic marker GFAP (red) staining in the hippocampus on POD14, scale bar: 100 um. **B-E**: histogram show the quantified analysis of total C3 intensity (**B**), the percentage of C3^+^ signal occupied within GFAP^+^ area (**C**), total GFAP intensity (**D**), and the average of GFAP^+^ cell size (**E**), Two-way ANOVA with Bonferroni’s multiple comparison test with n = 4 mice per group. **F**: heatmap of cytokine mRNA expression on POD 14, Two-way ANOVA with Bonferroni’s multiple comparison test with n = 6–7 mice per group; * P < 0.05 for LAP + AAV9-GFP versus SEVO + AAV9-GFP, # p < 0.05 for LAP + AAV9-C3shRNA versus LAP + AAV9-GFP. Data is presented as mean ± SEM, * P < 0.05, **P < 0.01, ***P < 0.001. POD = post-operative day, SEVO = sevoflurane, LAP = laparotomy, AAV9-GFP = AV9 viral vector contains GFP sequence, AAV9-C3 shRNA contains C3 shRNA sequence and GFP sequence

Since C3 has been shown to mediate pro-inflammatory responses, we also evaluated the postoperative change in astrocytes and in cytokines after C3 inhibition. Firstly, GFAP and C3 double staining showed that AAV9-mediated C3 suppression occurred only within hippocampal astrocytes of postsurgical mice but not in those exposed to sevoflurane only (Fig. 7A-C). However, C3 inhibition did not affect activated astrocytes, indicated by GFAP^+^ intensity, cell size and cell number (Fig. 7A, D-E). In addition, expression profiles of cytokines showed a decrease in MCP-1 induced by C3 inhibition only (Fig. 7F). Taken together, inhibition of C3 by AAV9 approach did not affect the activation state of reactive astrocytes and expression of various cytokines in the hippocampus. The results suggested that C3 was not the major factor to regulate neuroinflammation orchestrated by cytokines.

### Inhibition of C3 By AAV-C3shRNA Attenuated Microglia to Phagocytose Synapses

**Fig. 8:**
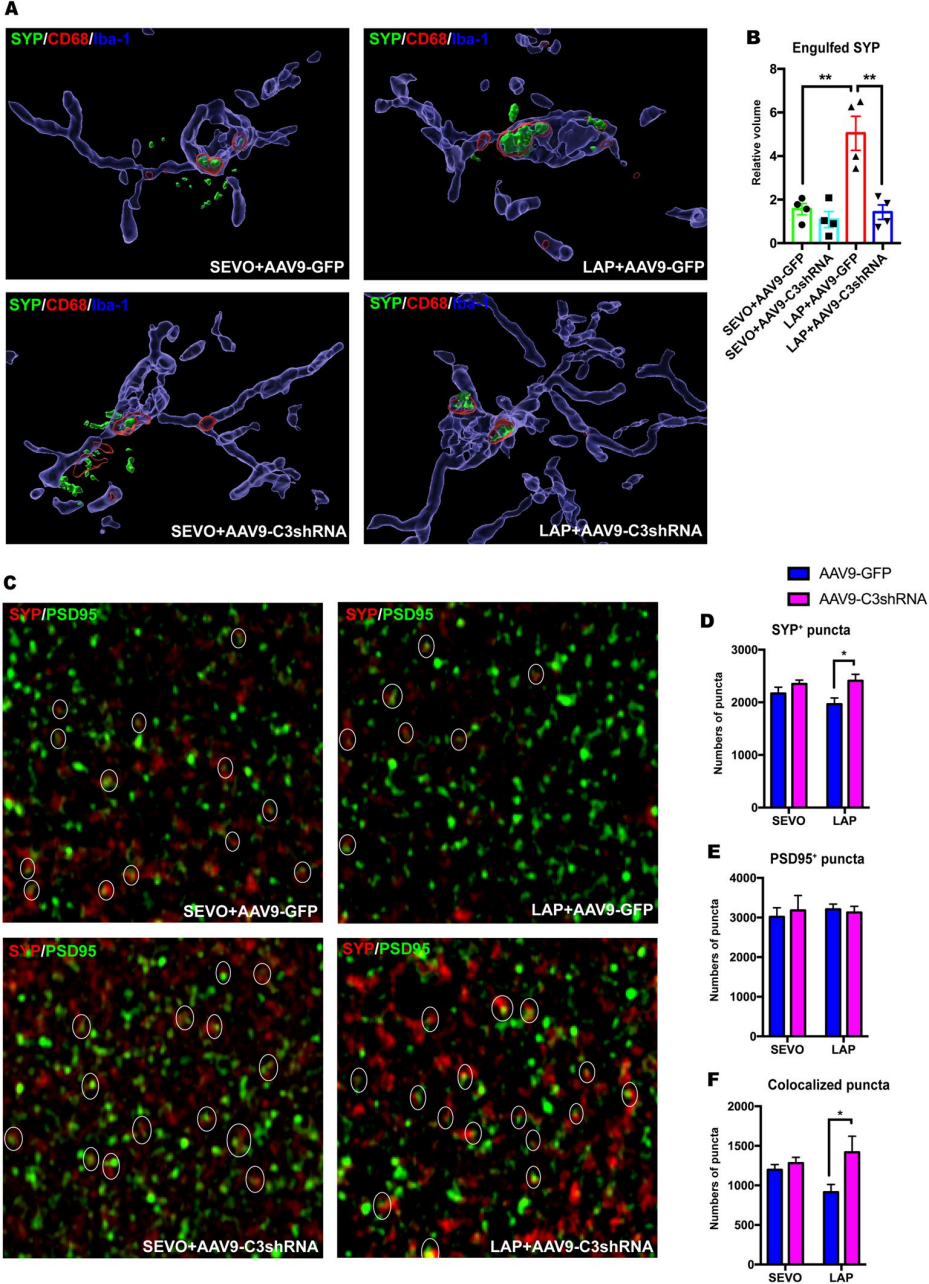
Knockdown of C3 prevented microglia-mediated engulfment of synapses and preserved synapse in the hippocampus.** A**: representative image of 3D reconstruction and surface rendering of pre-synaptic marker SYP (green), lysosome marker CD68 (red) and microglial marker Iba-1 (blue) depicting microglial engulfment of synapses; **B**: histogram of engulfed SYP volume showing that C3 knockdown can attenuate laparotomy-induced microglial engulfment of synapses on POD14. **C**: representative image of SYP^+^ (red) pre-synaptic and PSD95^+^ (green) post-synaptic puncta. **D-F**: histogram of puncta numbers showing that C3 knock-down augmented the number of SYP^+^ puncta (**D**), and colocalized puncta (**F**), with no significant difference of PSD95^+^ puncta (**E**). Two-way ANOVA with Bonferroni’s multiple comparison test with 6 cells per mouse, n = 4 mice per group. Data was presented as mean ± SEM, * P < 0.05, **P < 0.01, ***P < 0.001. PSD95: postsynaptic density protein 95, SYP: synaptophysin, SEVO = sevoflurane, LAP = laparotomy, AAV9-GFP = AAV9 control viral vector containing short non-sense sequence and GFP sequence, AAV9-C3 shRNA = AAV9 viral vector containing C3 shRNA sequence and GFP sequence

Another role of complement C3 in the CNS is to mediate engulfment (phagocytosis) of synapses by microglia. To further investigate the impact of C3 activation on hippocampal synapses, we assessed phagocytic activity of microglia in the hippocampus by performing high-resolution confocal imaging and 3D reconstruction analysis. Consistent with C3 activation, we found a significant augmentation of SYP^+^ presynaptic terminals engulfed within microglial lysosomes of postsurgical mice pretreated with AAV9-GFP compared to their SEVO + AAV9-GFP controls, which was significantly attenuated by AAV9 deliver of C3 shRNA in postsurgical mice **(**Fig. 8A-B). Then, we investigated the change of CD68 expression and activation of microglia in the hippocampus (Suppl. Fig. 3A). The results showed that a higher intensity of CD68 in the LAP group was decreased by C3 inhibition (Suppl. Fig. 3B), while the increase of Iba-1 intensity in the LAP group was not changed by C3 inhibition (Suppl. Fig. 3C). However, the size of Iba-1 positive cells in the postsurgical hippocampus was significantly reduced by C3 inhibition (Suppl. Fig. 3D). These findings suggested that C3 inhibition could not completely reverse activation of microglia, but alleviate phagocytic function in microglia at least.

We next evaluated loss of synapses in the hippocampus and identified that knockdown of C3 by AAV9-shRNA approach significantly rescued the loss of SYP^+^ presynaptic terminals and SYP^+^/PSD95^+^ colocalized synaptic puncta in the postsurgical mice, which was consistent with C3-associated elimination of synapse by microglia (Fig. 8C-F). However, no significant change of PSD95^+^ postsynaptic terminals by either surgery or C3 inhibition was found. The results suggest that activation of C3 after surgery promoted engulfment of synapses by microglia, and suppression of C3 reduced phagocytosis of synapses by microglia. The results also explain why sustained activation of microglia could be found two weeks after post-surgery even though expression of cytokine returned to basal level.

## Discussion

Our previous studies have shown that laparotomy leads to systemic inflammation and neuroinflammation, phosphorylation of tau, production of β-amyloid peptides, and even cognitive dysfunctions (Huang et al. 2018a, b; Chu et al. 2022). While it has long been considered that systemic pro-inflammatory cytokines modulate neuroimmune responses of astrocytes and microglia, our previous findings have shown that the levels of cytokines in the periphery and in the brain are essentially returned to basal levels after 2 weeks of laparotomy (Huang et al. 2018a, b). However, both astrocytes and microglia retain in the activated state. While there are several hypotheses to explain sustained activation of microglia, we found that complement C3 plays significant roles in sustained activation of microglia and cognitive dysfunctions. By examining gene expression, C3 was the major pro-inflammatory factor retained at high levels 2-week after laparotomy. By using AAV-C3-shRNA to knockdown C3, cognitive dysfunctions were attenuated and microglia no longer phagocytose pre-synaptic density protein. However, C3 did not affect reactive state of astrocytes. Our findings are among the first to show the role of C3 in retaining neuroimmune responses after systemic immune responses. Although general clinical practice considers that suppression of body immune responses by anti-inflammatory medicine is sufficient, our previous findings demonstrate that neuroimmune responses may require special care to minimize the harmful effects of neuroinflammation even systemic inflammation is halted. Our current findings suggest that complement C3 is the most suitable molecular target to attenuate sustained activation of microglia and cognitive dysfunctions.

Neuroinflammation has been regarded as a vital component in the pathogenesis of perioperative neurocognitive disorders (Safavynia and Goldstein 2018; Luo et al. 2019). Previously, we observed early and rapid changes of cytokines, prolonged activation of microglia and cognitive impairment in the postsurgical mice, changes that could be minimized by anti-inflammation drugs (Chu et al. 2018; Huang et al. 2018b). However, it is unclear how the rapid rise and fall of cytokines result in prolonged cognitive dysfunctions and why there is sustained activation of microglia and the role they play. In addition to activation of microglia and rapid increases of IL-1β and TNF-α in the hippocampus, here we also reported reactive astrocytes and upregulation of complement C3. Unlike the temporary changes of cytokines, a delayed production of C3 coincides more closely with the appearance of postoperative cognitive deficits. Inflammatory factors such IL-1β, TNF-α, and C1q can induce reactive astrocytes, a major resource of cerebral C3 (Ben Haim et al. 2015; Liddelow et al. 2017; Hou et al. 2020). Thus, temporary rise of IL-1β and TNF-α on POD 1 may account for the observed activation of neurotoxic astrocytes and upregulation of C3 in this model.

It has been generally considered that C1q and C3 are upregulated and promoted elimination of synapses by microglia in the pathological brain (Stephan et al. 2012; Tenner et al. 2018). Neurons and activated microglia are the main sources of C1q in the brain (Veerhuis et al. 2011; Chen et al. 2022b), accumulated burden of Aβ plaques and disrupted synaptic structure induced an increase of C1q, which then tags the affected synapses (Fonseca et al. 2004; Dejanovic et al. 2018; Carvalho et al. 2019; Spurrier et al. 2022). Accumulation of C1q on synapses further triggered the activation of downstream molecule C3 and resulted in synaptic elimination by microglia via its complement receptors CR3 or C3aR (Fonseca et al. 2004; Hong et al. 2016; Litvinchuk et al. 2018). However, C3 is not only the downstream molecule of the classical pathway, C3 itself can directly initiate activation of the complement cascade via the alternative pathway (Propson et al. 2021; Chen et al. 2022b). In addition, previous findings on demyelinating diseases demonstrated that C3 can bind to myelin directly and mediate engulfment of synapses by microglia (Sospedra and Martin 2005; Werneburg et al. 2020). In this study, only C3 but not C1q was increased after laparotomy, which implicates activation of microglia to perform phagocytosis of synapses. In agreement with this line of thinking, we also demonstrated that knockdown of C3 can successfully preserve synapses in the postsurgical hippocampus and cognitive functions. Thus, it is C3 that stimulates microglia phagocytosis even in the low level of C1q, which leads to phagocytosis of tagged or non-tagged synapses. Besides, our data also showed that suppression of C3 decreased microglial cell size and CD68 expression, but did not affect astrocyte activation or expression of cytokines, which suggested that C3 acts as the major factor to sustain activation of microglia, which is independent of astrocytes and cytokines.

Functioning astrocytes are not only essential for brain health but also play a significant role in neuroinflammation (Ben Haim et al. 2015; Dallérac and Rouach 2016). They can become reactive in response to a variety of pathological stimuli, including tissue injury, infection, ischemia, and also in neurodegenerative conditions (Ben Haim et al. 2015; Dallérac and Rouach 2016; Liddelow et al. 2017; Clarke et al. 2018). Astrocytic reactivity can also be induced by cytokines, aberrant proteins, or an imbalance in neurotransmitters and is initially characterized by hypertrophy and GFAP overexpression (Wilhelmsson et al. 2006; Ben Haim et al. 2015). Reactive astrocytes could be classified into two phenotypes: the neurotoxic (or A1) and the neurotrophic (or A2) astrocytes (Ben Haim et al. 2015; Liddelow et al. 2017). A1 astrocytes upregulate various complement components and C3 is considered as one of the markers of A1 astrocytes (Liddelow et al. 2017). These neurotoxic astrocytes are present in multiple neurodegenerative diseases, such as Alzheimer’s disease and multiple sclerosis (Liddelow et al. 2017; Li et al. 2020). Our data showed that hypertrophic astrocytes with higher GFAP expression are found in the hippocampus of mice from the laparotomy group and further identified as A1 astrocytes by overexpression of pan and A1 markers, indicating the reactive astrocyte is neurotoxic.

There are several limitations of the experimental model used in this study that will restrict its generalizability as we used only young male mice and examined only the hippocampal region. We acknowledge that perioperative neurocognitive disorders occur more in the elderly and is not gender specific, although the data is supportive of our hypothesis, more studies being directed at aged animals and both sexes are needed in the future. Another major limitation is the knock-down of cerebral C3 by general anti-C3 shRNA viral vector rather than specific knock-down of astrocytic C3. This will likely result in the suppression of neuronal or glial C3 could affect cognitive behaviors. Additionally, there is an age dependent variation to C3 expression (Veerhuis et al. 2011; Luchena et al. 2018) but we only examine young adult mice so the results may not be applicable to other age groups. Furthermore, we did not perform any experiments involving overexpression of C3 and evaluate its effect. Further studies using conditional C3 knock-down viral vector and C3 overexpression mice could be helpful to confirm the protective effects of C3 knock-down in perioperative neurocognitive disorders.

Taken together, our results demonstrated that complement C3 was the major pro-inflammatory factor keeping at the high levels in the hippocampus 2 weeks after laparotomy, while most of cytokines returned to basal levels. Inhibition of C3 prevented phagocytosis of synapse by microglia and attenuated cognitive dysfunctions without affecting activation of astrocytes and expression of cytokines. These findings demonstrated that complement C3 plays significant roles in retaining neuroimmune responses after systemic immune responses, which originally explained how sustained activation of microglia and cognitive dysfunctions happens while various cytokines returned to basal levels. This gives further supports to develop a therapeutic strategy that targets complement C3 to attenuate sustained activation of microglia and cognitive dysfunctions.

## Supplementary Information

Below is the link to the electronic supplementary material.

Supplementary file1 (DOCX 3192 KB)

## Data Availability

The datasets generated or analysed during the current study are available in the HKU Data Repository, figshare, DOI 10.25442/hku.21399510. The sequencing data that support the findings of this study have been deposited in GEO with the accession number: GSE199318.
