# Supplementary material for: Complement C3 From Astrocytes Plays Significant Roles in Sustained Activation of Microglia and Cognitive Dysfunctions Triggered by Systemic Inflammation After Laparotomy in Adult Male Mice
Source: J Neuroimmune Pharmacol. 2024 Mar 1;19(1):8. doi: 10.1007/s11481-024-10107-z (PMC10907447; doi:10.1007/s11481-024-10107-z)
Supplement: Supplementary file 1 — Supplementary file1 (DOCX 3192 KB) [file 11481_2024_10107_MOESM1_ESM.docx]

**Supplementary Materials**

**Materials and Methods**

**Behavioral Tests**

**Open field (OF) test:** The OF test is a classical behavior test used to evaluate locomotor activity and the level of anxiety in rodents that relies on their spontaneous and exploring activities. After 30 min habituation in a dimly lit behavioral room, the mouse was placed into the white enclosed gridded arena with an area of 40 cm X 40 cm X 40 cm and was allowed to freely explore for 10 min as previously described (Liu et al., 2022). The arena was divided equally into 25 square zones with the middle 9 zones demarcated as the central area. The total exploration time spent in the central area is indicative of the anxiety level of mice, and the total exploration distance and total frequency of grid lines crossing being an indicator of locomotor activity.

**Novel objection recognition (NOR) Test:** The NOR test assesses the rodent’s ability to recognize novel objects in a controlled environment and is a measure of recognition memory, which was performed according to previously described protocol with minor modification (Liu et al., 2022). Twenty-four hours prior to conducting the test, the mice were habituated in an empty open field arena with evenly lit conditions. On Day 1 for familiarization, two identical objects (A_1_+ A_2_) were placed in opposite, but symmetric sides of the arena and the mouse was allowed to freely explore the two objects for 10min. On Day 2 for discrimination, one of object (A) was replaced with a novel object (B), and the mouse was placed back into the same arena and allowed to explore the objects (A + B) for 10 min, and their behavior was video recorded. An interaction with an object was defined by the mouse nose pointing at and/or touching the object. The discrimination index is the ratio of the exploration time with novel object (B) over the sum of the total exploration time with objects (A + B).

**Forced alternation Y-maze test:** This Y-maze test is used for determining hippocampus-dependent memory function as described before (Huang et al., 2018). The testing device contains two dark arms that can deliver a small electric shock and one white safe, i.e., shock free arm with a transparent cover. During preoperative training, the mice was allowed to habituate in the Y-maze for 10 min for free alternations. After that, the mouse was placed in one of the ends of a dark arm, and electric shocks (2 Hz for 10 s at 40 ± 5 V) were applied to force the mouse to enter the white arm and to stay there for 30 s, which then is regarded as a correct choice. Training was considered successful if the mouse makes 9 consecutive correct choices. During the testing phase, the mouse was placed alternately in one of the dark arms on ten occasions. The number of incorrect choices and the time taken (latency) to enter the white arm were recorded to evaluate the learning and memory functions of mice.

**Total mRNA extraction and RT-PCR**

After behavioral tests, the mice were euthanatized by CO_2_ asphyxiation and subjected to trans-cardiac perfusion with cold saline. The hippocampi were dissected and stored at -80^0^C. The right side of hippocampus was used for total mRNA extraction and the left side of hippocampus were used for protein extraction. Total mRNA was isolated by using RNAiso plus (Takara, Japan, Cat. # 9109). Only those mRNA samples with the ratio of OD260/280 being between 1.8-2.0, were qualified to be converted into complementary DNA (cDNA) by using the PrimeScript^TM^ Master Mix Kit (Takara, Japan, Cat. # RR036B). The expression of complement factors, cytokines and other genes were assessed by real-time PCR using TB green premixed reagent (Takara, Japan, Cat. # RR820A). The primers used in this current study are listed in Suppl. Table 1.

**Western Blot analysis**

The dissected hippocampi from the left hemispheres were homogenized by RIPA lysis buffer containing proteases and phosphatase inhibitors. The protein supernatants were collected and measured by BCA assay. Denatured proteins were subjected to 8-12% SDS-PAGE gel electrophoresis and transferred onto PVDF membranes (Bio-rad, USA). After blocking with 10% non-fat milk solution, the membranes were incubated with specific primary antibodies: complement C3 (1:500, Invitrogen, USA, Cat. # PA5-21349), β-actin (1:1000, Sigma, USA, Cat. # A5441), β-tubulin (1:1000, CST, USA, Cat. # 2128) overnight at 4 ^0^C and subsequently incubated with HRP-conjugated secondary antibodies (1:20000, Jackson, USA, Cat. # 115-035-003, # 111-035-003) for 2 h at room temperature. The protein bands were visualized by enhanced chemiluminescence reagents and captured by ChemiDoc^TM^ Touch Imaging System (Bio-Rad, USA). The intensity of bands was quantified by ImageJ.

**Immunofluorescent Staining**

Harvested brains were immersed in 4% PFA at 4^0^C for 24 h, followed by 20%-30% sucrose solution dehydration. The tissue was then embedded in O.C.T and after it solidified, the brains were cut into 20um-thickness coronal cryosection. The brain sections were blocked in Phosphate buffer saline (PBS) with 5% bovine serum albumin (BSA, Sigma, USA) and 0.3% Triton^TM^-100 (Sigma, USA, Cat. # 93433) at room temperature for 1h, then incubated with specific primary antibodies: GFAP (1:800 Sigma, USA, Cat. # G3893), Iba-1 (1:500 Wako, Japan, Cat. # 016-26721), CD68 (1:200 Invitrogen, USA, Cat. # MA5-16674), C3 (1:200 Abcam, USA, Cat. # ab11862), Synaptophysin (SYP, 1:500 SYSY, Germany, Cat. # 101004), PSD95 (1:500 SYSY, Germany, Cat. # 124003) overnight at 4^0^C. After rinsing with PBS, the sections were incubated with secondary antibodies conjugated with fluorochrome for 2 h at room temperature and mounted with Prolong Gold mounting medium containing DAPI (Thermo Fisher Scientific, USA, Cat. # P36931). The Z-stacks of fluorescent signals were captured at 0.3um steps by LSM 880 confocal microscope (Zeiss) equipped with airyscan detector, diode lasers (405 nm, 488 nm, 568 nm and 647 nm) and Zen black acquisition software.

**Microglial engulfment analysis**

Microglial engulfment analysis was performed according to the previously published protocol with slight modification (Werneburg et al., 2020). Brain sections containing hippocampal region in mouse were stained with SYP, Iba-1 and CD68 antibodies and imaged by a Zeiss LSD880 confocal microscope and Zeiss acquisition software. 6-8 cells from randomly chosen 63x field of view within hippocampal region of each mouse were acquired with 30-50 z stack steps at 0.3 um interval using Airyscan mode, followed by airyscan processing performed by Zeiss software. Engulfment analysis of individual cells was processed by IMARIS. 3D rendering surface of each channel was reconstructed respectively. Only the volume of SYP^+^ signal restricted within CD68-positive microglial lysosome were measured as engulfed SYP. Unbiased quantification of all images was performed blind to genotype or treatment of animals.

**Synaptic puncta analysis**

As for synaptic puncta analysis, the staining and analysis procedure was modified according to the previously described protocol (Ippolito and Eroglu, 2010). Briefly, the double immunofluorescent staining of SYP and PSD95 was applied to brain sections with hippocampal region. 2-3 randomly selected 63x field of view within hippocampal region were imaged 12 z stack steps at 0.3um interval in both 568 nm and 647 nm channel using Zeiss LSD880 confocal microscope and Zeiss acquisition software. Maximum intensity projection (MIP) was generated from 3 serial sections yielding a total of 4 MIPs, followed by synapse quantification by Image J software installed with “synaptic analyzer” plugin.

**Flow cytometry**

After mice were euthanized and quickly decapitated, the hippocampi were dissected in cold HBSS solution. After the removal of the meninges and blood vessels, the hippocampus was minced into small pieces and digested with Collagenase IV (5 ug/ml, Germany, Cat. # LS004188) with DNase I (100 ug/ml, Sigma, USA, Cat. # DN25) for 30 min at 37^0^C. After gentle pipetting and centrifuging at 300 g, the pellet was resuspended with PBS and passed through a 70-um strainer. The single cell suspension was centrifuged again for 10 min at 300 g. The pellet was resuspended by 30% percoll and centrifuged without braking at 700 g 30 min at 20^0^C to get rid of the myelin. The cell pellet was then resuspended in 100 ul of a blocking buffer consisting of PBS with 0.5% BSA.

All flow cytometry staining was performed at 4^0^C. 2 ul of Fc receptor blocking reagent (Biolegend, USA, Cat. # 156604) was added and incubated for 10 min. Subsequently, the cell suspension was incubated for 20 min with anti-mouse GLAST primary antibody (conjugated to APC, 1:50, Miltenyi, Germany, Cat. # 130-123-555). To distinguish between live and dead cells, 3 mM of DAPI stain was add and incubated for 10min. After washing with the blocking buffer, the cell pellet was resuspended in 500 ul of blocking buffer.

A FACS Aria™ Fusion Flow Cytometer (BD biosciences, USA) was used for cell sorting. DAPI^-^/GLAST^+^ cells as live astrocytes were sorted into separated tubes and consistently kept in a 4^0^C environment. DAPI^-^/GLAST^+^ sorted cells were centrifuged at 6000 g for 5 min at 4^0^C. Total RNA of the sorted cell pellet was extracted by using the RNeasy Micro kit (Qiagen, Germany, Cat. # 74004), genomic DNA was removed by DNase digestion. The concentration and purity were measured by Nanodrop and Agilent 2100 bioanalyzer.

**RNA-sequencing**

The total RNA of sorted GLAST^+^ cells pooled from 6 different hippocampi were considered as one biological replicate, and for each group (viz SEVO and LAP), 3 biological replicates were used to perform the RNA-seq. The quality of the total RNA yield was assessed by Agilent2100. Total RNA was purified, fragmented and reverse transcribed into cDNA, followed by poly-A tail ligation and enrichment. Sequencing was performed on DNBSEQ platform developed by Beijing Genomics Institute. Before analysis, 4GB clean reads from each sample were obtained after filtering and the removal of low-quality reads and mapped to the reference genome and reference gene by using the HISAT and Bowtie databases separately. The differential expression genes (DEGs) were defined by DEGseq with the settings: |log2 (foldchange)|≥1 and Q value ≤0.05, followed by Gene Ontology and Kyoto Encyclopedia of Genes and Genomes (KEGG) pathway enrichment analysis. The expression level was visualized by fragment per kilobase million (FPKM).

**Table 1: The list of primers used in this current study.**

| GFAP forward: | CAG AGG AGT GGT ATC GGT CTA A |
| --- | --- |
| GFAP reverse: | GAT AGT CGT TAG CTT CGT GCT T |
| C3 forward: | CAG CTT CAG GGT CCC AGC TA |
| C3 reverse: | CTC CAG CCG TAG GAC ATT GG |
| C1q forward: | TCA CCA ACC AGG AGA GTC CA |
| C1q reverse: | CAC CTG AAA GAG CCC CTT GT |
| IL-1β forward: | CCT CCT TGC CTC TGA TGG |
| IL-1β reverse: | AGT GCT GCC TAA TGT CCC |
| IL-6 forward: | TTC ACA AGT CCG GAG AGG AG |
| IL-6 reverse: | TCC ACG ATT TCC CAG AGA AC |
| TNF-α forward: | CCC CAG TCT GTA TCC TTC T |
| TNF-α reverse: | ACT GTC CCA GCA TCT TGT |
| IL-10 forward: | CCA AGC CTT ATC GGA AAT GA |
| IL-10 reverse: | TTC TCA CCC AGG GAA TTC AA |
| MCP-1 forward: | AGC TGT AGT TTT TGT CAC CAA GC |
| MCP-1 reverse: | GAC CTT AGG GCA GAT GCA GT |
| IL-17 forward: | CTG TGT CAA TGC GGA GGG AA |
| IL-17 reverse: | CCC ACC AGC ATC TTC TCG AC |
| CD44 forward: | TTG TCA ACC GTG ATG GTA CTC |
| CD44 reverse: | GCT GCT GAC ATC GTC ATC TAT |
| Serpina3n forward: | CGA AAC TGT ACC CTC TGA CTG |
| Serpina3n reverse: | GGG TTG GCT ATC TTG GCT ATA A |
| Cxcl10 forward: | GGC CAT AGG GAA GCT TGA AA |
| Cxcl10 reverse: | CAG ACA TCT CTG CTC ATC ATT CT |
| Ggta1 forward: | TGC TTG TCT GGG CCA CTA TC |
| Ggta1 reverse: | TCT CCA GCC TCC TAA GAC ACA |
| Serping1 forward: | GCC CAT GAT GAG TAG CGT AAA |
| Serping1 reverse: | GCC CAT GAT GAG TAG CGT AAA |
| Fkbp5 forward: | GAA AGG CGA GGG ATA CTC AAA |
| Fkbp5 reverse: | CCA CAT CTC GGC AAT CAA ATG |
| H2-T23 forward: | GAT CTC TAA GCA CAA GTC AGA GG |
| H2-T23 reverse: | CAT TTC CCA GCC GTA GGT ATC |
| EMP1 forward: | CTG TGT ATC CTG GTT GGA GTG |
| EMP1 reverse: | TCC AGG TCA GGA TGA AAC AAT AG |
| S100a forward: | GGC TTC CAG AGC TTT CTA TCA C |
| S100a reverse: | CCC TTC TGC TTC ATG TTT ACT ACA |
| CD209 forward: | AGT GAC TCC ACA GAA GCC AAG |
| CD209 reverse: | CCA AGA ACA GGA AGG AGA GCA |
| IL-1rn forward: | GGC AGT GGA AGA CCT TGT GT |
| IL-1rn reverse: | ATG AGC TGG TTG TTT CTC AGG T |
| Pcdhga3 forward: | GCT TTA ATC CAT CGT GGG AAT CAG |
| Pcdhga3 reverse: | GGA GCC TTT GTC CAG TTC CT |
| F8 forward: | GCA TGG AGT TGA TGG GCT GT |
| F8 reverse: | GAG GTC GCC AGG CAT TAG TC |
| Jpt2 forward: | AGA GGG TTT GTC ATG CGT GTG |
| Jpt2 reverse: | GGA CCA GAG CAT CCC AGT TTA |
| Sebox forward: | TGG CCT TGT AGC CCA TAG GA |
| Sebox reverse: | AGG ACC AGG ATG CCG AGT TT |
| Zfp131 forward: | TGG TTT GGT TTC TTG TTG TAG GG |
| Zfp131 reverse: | GTC CTG CTC CCG TTG TTC AT |
| Topbp1 forward: | TCG GGC TCC ACC TAG TTC A |
| Topbp1 reverse: | CTC TCT TAT CTT CAA TGC TTC CTC A |
| GFP forward: | GCA TCG ACT TCA AGG AGG AC |
| GFP reverse: | GAA CTC CAG CAG GAC CAT GT |

Huang C, Chu JM, Liu Y, Chang RC, Wong GT (2018) Varenicline reduces DNA damage, tau mislocalization and post surgical cognitive impairment in aged mice. Neuropharmacology 143:217-227.

Ippolito DM, Eroglu C (2010) Quantifying synapses: an immunocytochemistry-based assay to quantify synapse number. Journal of visualized experiments : JoVE.

Liu Y, Chu JMT, Ran Y, Zhang Y, Chang RCC, Wong GTC (2022) Prehabilitative resistance exercise reduces neuroinflammation and improves mitochondrial health in aged mice with perioperative neurocognitive disorders. Journal of neuroinflammation 19:150.

Werneburg S, Jung J, Kunjamma RB, Ha SK, Luciano NJ, Willis CM, Gao G, Biscola NP, Havton LA, Crocker SJ, Popko B, Reich DS, Schafer DP (2020) Targeted Complement Inhibition at Synapses Prevents Microglial Synaptic Engulfment and Synapse Loss in Demyelinating Disease. Immunity 52:167-182.e167.

**Suppliementary Figures:**

**Suppl. Figure 1**

***
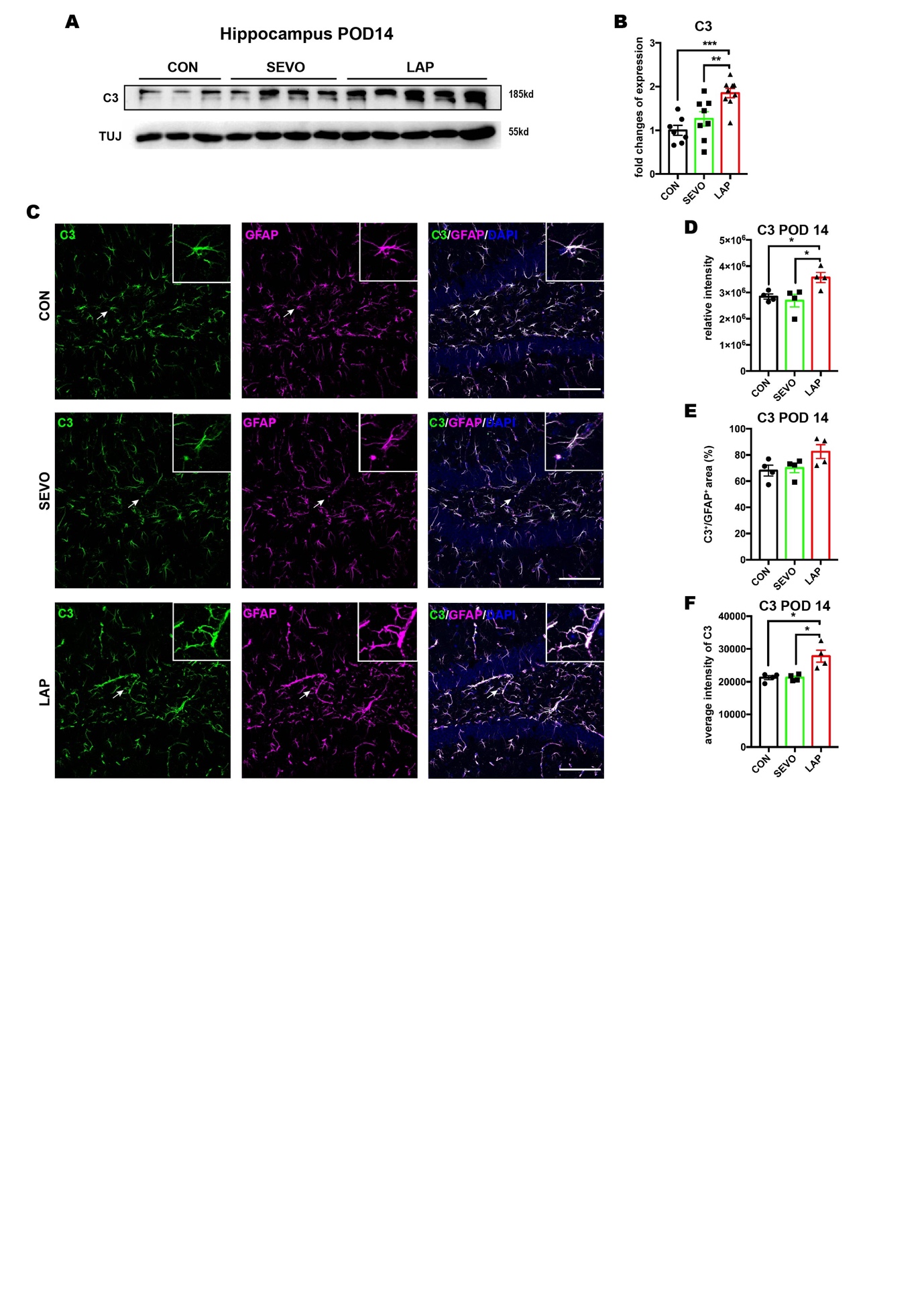
***

**Suppl. Figure 1: The increase of astrocytic C3 was also observed in postsurgical hippocampus on POD 14. A-B:**  Western blot analysis of C3 protein level in the hippocampus on POD 14, normalized to β-tubulin, n = 7-10 mice per group. **C:** representative confocal images depicting colocalization of C3 (green) and astrocytic marker GFAP (red) staining in the hippocampus on POD14, scale bar: 100um. **D-F:** histogram showing the quantified analysis of total C3 intensity (**D)**, the percentage of C3^+^ signal occupied within GFAP^+^ area (**E**), and average C3 intensity within GFAP^+^ cell (**F**), n = 3-4 mice/group, scale bar: 100um. Data is presented as mean ± SEM. One-way ANOVA with Tukey’s multiple comparisons test was performed for data analysis, * P<0.05, **P<0.01, ***P<0.001. POD = post-operative day, CON = control, SEVO= sevoflurane, LAP =laparotomy.

**Suppl. Figure 2**


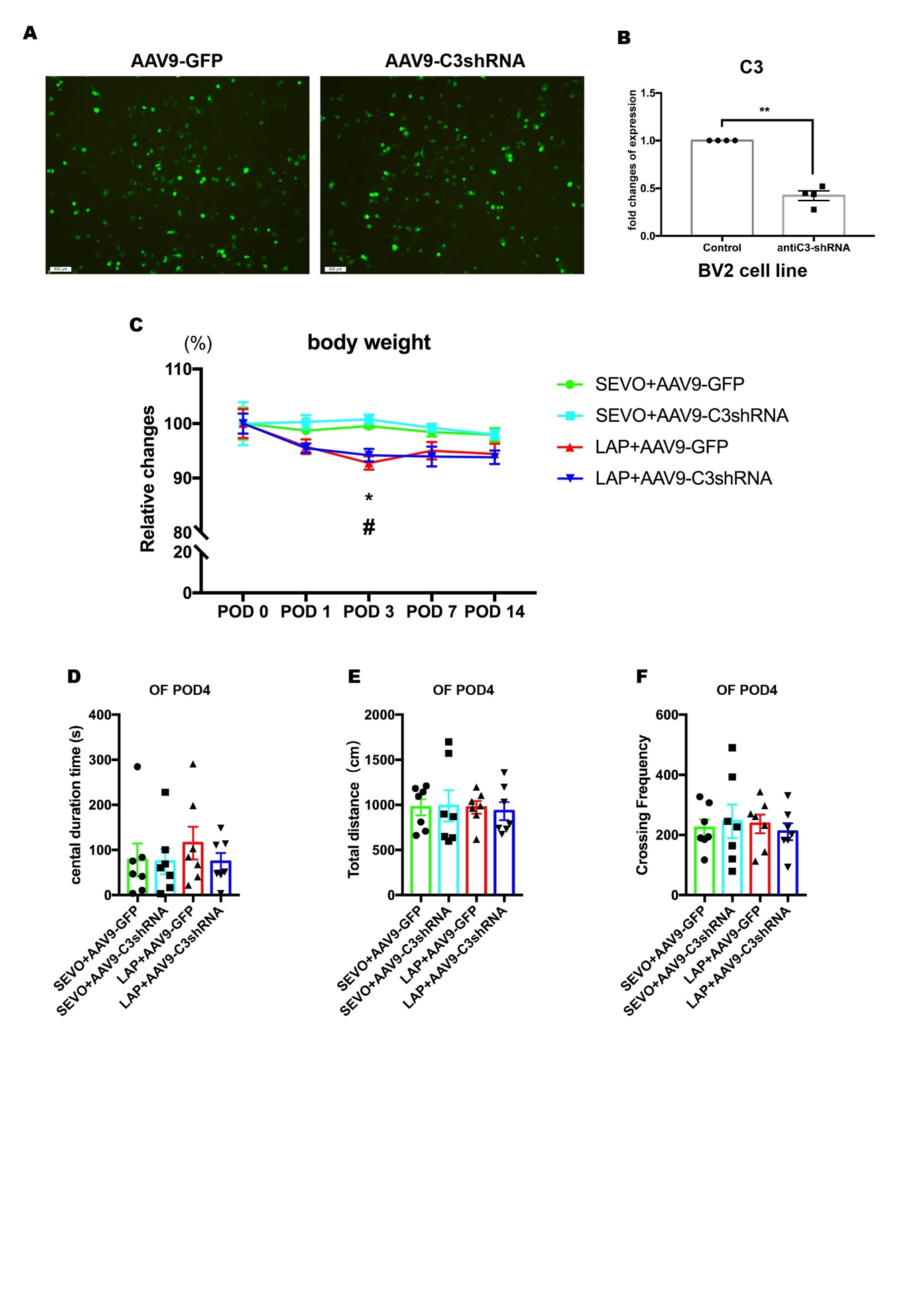


**Suppl. Figure 2: A:** Representative images of AAV9-C3shRNA transfection into BV2 cell line at 24h time point. **B**: Histogram shows that the C3 mRNA level was decreased by around 50% after 24h AAV9-C3shRNA transfection, measured by RT-PCR, control: BV2 cells treated by AAV9-GFP, antiC3-shRNA: BV2 cells treated by AAV9-C3shRNA. **C**: The changes in body weight at different timepoints during the postoperative period. Two-way repeated ANOVA with Tukey’s multiple comparison test with n =7 mice per group. SEVO+AAV9-GFP vs LAP+ AAV9-GFP: * P<0.05. SEVO+AAV9-C3shRNA vs LAP+AAV9-C3shRNA: #P<0.05. **D-F**: Open field test on POD4, the duration time in the central area (**D**), Total distance (cm) during 10 mins observation (**E**), the frequency of grid crossing (**F**). Two-way ANOVA with Bonferroni’s multiple comparison test with n = 7 mice per group. POD = post-operative day, SEVO = sevoflurane, LAP =laparotomy, AAV9-GFP = AV9 viral vector contains GFP sequence, AAV9-C3 shRNA contains C3 shRNA sequence and GFP sequence.

**Suppl. Figure 3**


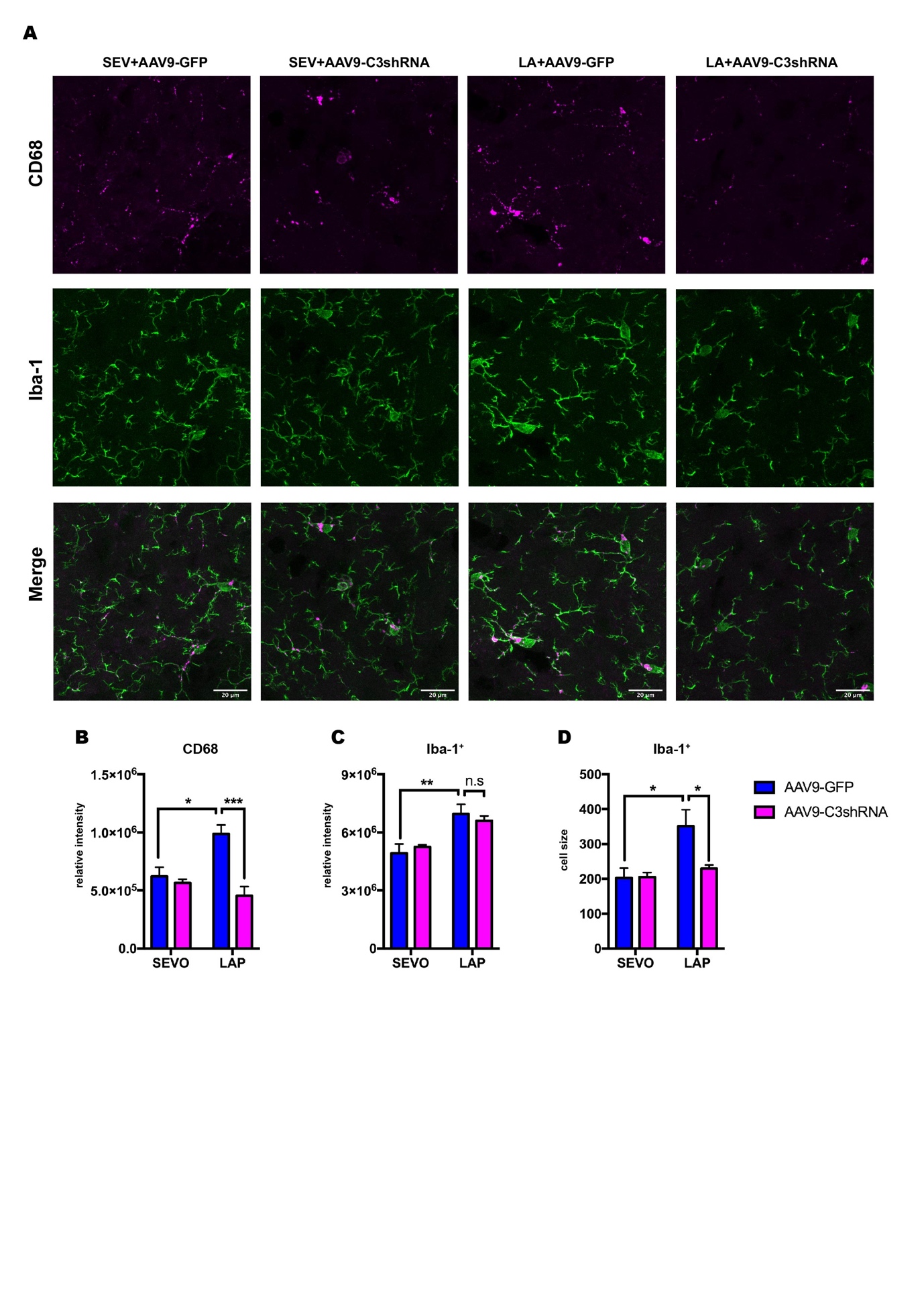


**Suppl. Figure 3: Inhibition of C3 by AAV-C3shRNA affected microglial cell size and CD68 expression. A:** Representative confocal images depicting colocalization of CD68 (magenta) and microglial marker Iba-1 (green) staining in the hippocampus on POD14, scale bar: 20 um. **B-D**: histogram show the quantified analysis of the CD68 intensity (**B**), the Iba-1 intensity (**C**), and the average of Iba-1^+^ cell size (**D**), Two-way ANOVA with Bonferroni’s multiple comparison test with n = 4 mice per group. SEVO = sevoflurane, LAP =laparotomy, AAV9-GFP = AV9 viral vector contains GFP sequence, AAV9-C3 shRNA contains C3 shRNA sequence and GFP sequence.

**Suppl. Figure 4**


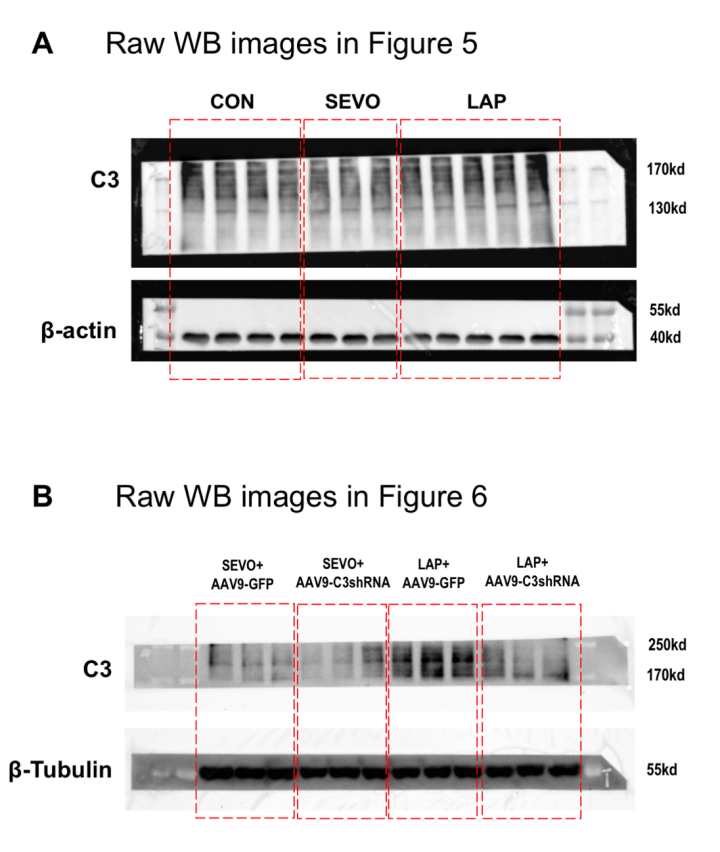


**Suppl. Figure 4: A:** The raw blot images in Figure 5**, B:** The raw blot images in Figure 6**.**
